# Predicting bacterial-mediated entomopathogenicity through comparative genomics and statistical modeling

**DOI:** 10.1128/spectrum.03108-25

**Published:** 2025-11-26

**Authors:** Daniela Yanez Ortuno, Melissa Y. Chen, Keegan McDonald, Allison Gacad, Juli Carrillo, Cara H. Haney

**Affiliations:** 1 Department of Microbiology and Immunology, The University of British Columbia 8166, Vancouver, British Columbia, Canada; 2 Faculty of Land and Food Systems, The University of British Columbia 8166, Vancouver, British Columbia, Canada; 3 Department of Biological Sciences, The University of Pittsburgh 6614, Pittsburgh, Pennsylvania, USA; University of Minnesota Twin Cities, St. Paul, Minnesota, USA

**Keywords:** fruit flies, *Drosophila melanogaster*, *Pseudomonas*, insecticidal activity, host-microbe interactions

## Abstract

Bacterial genomes encode vast functional diversity and have both beneficial and detrimental effects on insect hosts. While genotype-to-phenotype relationships are known for specific insecticidal genes on individual insect hosts, whether these mechanisms will be effective on a phylogenetically distinct insect host is not always known. To determine if known virulence genes are effective on a new host, we developed a method to merge existing mechanistic knowledge with *in vivo* tests on a small number of bacterial isolates to predict bacterial genes associated with entomopathogenesis. We used a model consisting of *Drosophila melanogaster* interactions with pathogenic and commensal genome-sequenced strains of *Pseudomonas* bacteria. We compiled a database of previously described insecticidal and biocontrol genes within the *Pseudomonas* genus and used comparative genomics to probe the distribution of these genes across *Pseudomonas* strains. We found natural variation in the presence of known insecticidal genes across the genus. We tested the insect-killing capacity of 13 *Pseudomonas* spp. strains against *D. melanogaster* and found natural variation in insecticidal activity. To identify bacterial genes associated with fly mortality, we employed two statistical models to correlate bacterial virulence with the presence of previously described insecticidal activity. To validate our predictions, we used a *P. aeruginosa* PAO1 transposon mutant library and identified eight operons that are necessary for killing *D. melanogaster*. We show that by combining existing literature with phenotyping a small number of strains, we identified both known and novel genes associated with insecticidal activity in *D. melanogaster*, using a rapid, scalable screening framework. More broadly, these findings illustrate a discovery pipeline for bacterial virulence mechanisms, accelerating the discovery of insect pest biocontrol mechanisms.

**IMPORTANCE:**

Bacteria with insecticidal properties offer a promising alternative to chemical pesticides, but identifying effective strains and their underlying mechanisms remains a challenge. Here, we used *Pseudomonas-D. melanogaster* as a model to develop a predictive framework for determining which known bacterial genes with insecticidal activity are effective in a new host. By integrating comparative genomics, statistical modeling, and experimental validation, we identified insecticidal genes that are effective in *D. melanogaster* and highlighted new candidates for future study, demonstrating the utility of our integrative modeling approach. Our findings show that genetic predictors of virulence vary across *Pseudomonas* phylogenetic groups, highlighting the potential for targeted biocontrol strategies. We also demonstrate that disrupting specific pathways significantly reduces insecticidal activity, confirming their role in bacterial virulence. As *Pseudomonas* strains are found in diverse environments, this approach may be broadly applicable for predicting insecticidal efficacy in other bacterial genera. By improving our ability to identify and engineer microbial biocontrol agents, this work advances sustainable pest management strategies and provides new tools for reducing reliance on conventional pesticides.

## INTRODUCTION

Insect pests significantly threaten global crop yields and food security. Pests account for 20%–40% of global crop losses annually, a challenge further intensified by climate change and growing pesticide resistance (1). For instance, many insect pests have developed insecticide resistance, sometimes to multiple insecticides with distinct modes of action, resulting in few existing treatments (2, 3). Furthermore, climate change has resulted in insect range expansion and increased crop stress, resulting in the need to rapidly develop new management tools (4–6). These examples highlight the pressing need for new and rapid pest management strategies to target existing and emergent pests.

Bacteria represent a promising source of insecticidal compounds, offering a sustainable alternative to conventional pesticides (7–9). Many bacterial species have evolved potent virulence mechanisms that enable them to kill insect hosts through diverse strategies, including toxin production, gut colonization, and immune suppression (10–13). However, the efficacy of bacterial insecticides is influenced by whether they function as generalists, capable of infecting multiple insect species, or specialists, which exhibit narrow host specificity. Generalists, such as *Photorhabdus* Tc toxins, exert broad insecticidal effects through systemic toxicity mechanisms, including pore formation and immune modulation (12, 14). In contrast, specialists, such as *Bacillus thuringiensis* (Bt) Cry toxins, rely on highly specific receptor interactions in the insect midgut, making them effective but also susceptible to resistance via single-gene mutations (15, 16).

While many insecticidal mechanisms have been extensively studied, their predictability across different bacterial strains and insect hosts remains poorly understood. For instance, *Bacillus sphaericus* effectively targets *Culex* and *Anopheles* mosquitoes but is ineffective against *Aedes aegypti* due to receptor-based resistance mechanisms (17). Similarly, bacterial colonization strategies, such as the Type VI Secretion System (T6SS), can play a crucial role in host interactions by modulating gut microbiota and delivering toxic effectors (18). These observations highlight the need for systematic and scalable approaches to predict bacterial insecticidal activity across insect hosts.

Existing reverse genetic approaches, such as genome-wide association studies (GWAS) or comparative genomics, have been successfully used to correlate genes or alleles with functional outcomes. These approaches have the benefit of being unbiased by *a priori* knowledge of gene function. However, they require subsequent filtering of candidates and assigning of functions, which requires substantial effort to determine if previously described insecticidal genes are among the candidates. As a result, we sought to develop a method that would constrain traditional GWAS or comparative genomics approaches by starting with a gene list of known insecticidal or antimicrobial functions, followed by comparative genomics and modeling.

In this study, we focused on *Pseudomonas* as a model bacterial system due to its genetic diversity, well-characterized virulence factors, and ability to colonize a wide range of environments and hosts (19–21). *Pseudomonas* strains exhibit insecticidal properties through diverse virulence factors such as pore-forming toxins, proteases, and secondary metabolites (22–24). *Drosophila melanogaster* serves as a valuable model for studying these interactions (25). Key virulence determinants in *Pseudomonas* have been identified in *D. melanogaster,* including Monalysin, a β-pore-forming toxin that damages the intestinal epithelium, and Fit toxins and cyclic lipopeptides like entolysin, which enhance pathogenicity under the control of the GacS/GacA regulatory system (26, 27). Despite this understanding of *Pseudomonas* virulence mechanisms, current methods for identifying bacterial insecticidal traits often rely on labour-intensive screening and mutant characterization, limiting the speed at which new candidates can be discovered.

To overcome these limitations, we developed a high-throughput, predictive framework that integrates existing literature with targeted experimental validation to systematically identify bacterial genes associated with insect killing. We constructed a database of insecticidal genes derived from literature on different bacteria and hosts and then we tested multiple *Pseudomonas* strains for their ability to kill *Drosophila melanogaster* and *Drosophila suzukii* via oral infection. Using two complementary statistical models, Cox Proportional Hazard and Random Forest Analysis, we correlated bacterial genetic profiles with insecticidal activity to identify genes predictive of virulence. This approach recovered several genes previously implicated in insecticidal activity in *D. melanogaster*, as well as others with limited or no prior functional validation in this host. Collectively, this work establishes a method for the rapid identification of bacterial biocontrol agents and offers a modeling framework that could, with host-specific refinements, be applied to diverse insect systems.

## RESULTS

### Natural variation of insecticidal and biocontrol genes in *Pseudomonas*

To investigate the distribution of insecticidal activity of *Pseudomonas* toward *D. melanogaster*, we first identified previously described insecticidal and biocontrol genes from the literature and used comparative genomics to map their distribution across the genus *Pseudomonas*. We primarily focused on genes in *Pseudomonas* that have been shown to have activity against any insects. We also included genes with biocontrol activity including anti-fungal, as these may have functions with toxicity against eukaryotic cells, as well as antibacterial activities that may help bacteria compete within a host environment. Then, we tested the functional distribution of insecticidal activity by testing the killing activity of a subset of strains in *D. melanogaster* infection assays.

**Fig 1:**
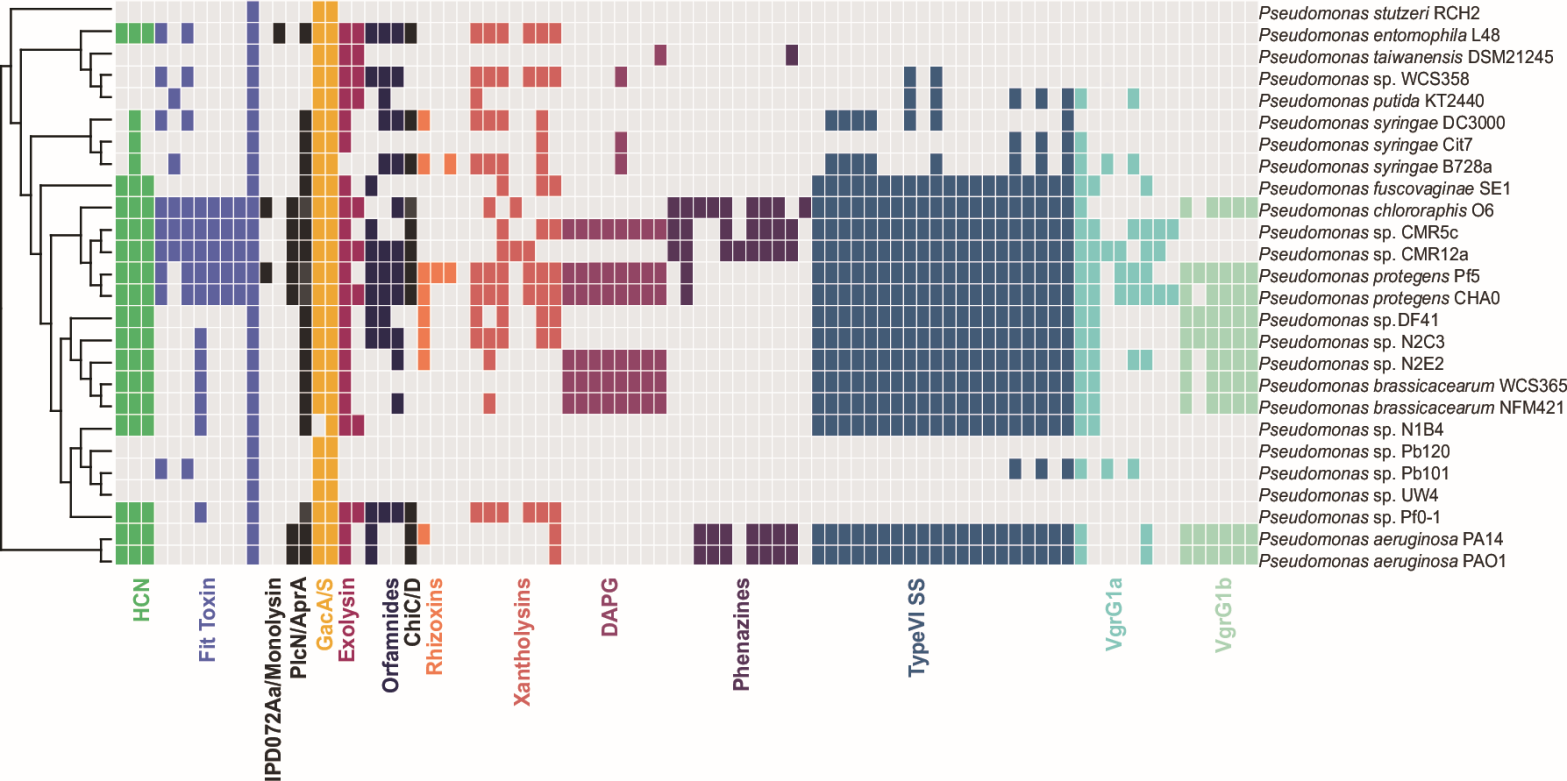
Distribution of insecticidal, biocontrol, and antifungal genes within the *Pseudomonas* genus. Colored squares represent the presence of homologous genes, while the absence is represented by white. The genus *Pseudomonas* is divided into five phylogenetic groups, *Pseudomonas aeruginosa, P. fluorescens*, *Pseudomonas putida, and Pseudomonas syringae*. Within the *P. fluorescens* group, subgroups include *P. chlororaphis* and *P. protegens*. The species tree was constructed using the PyParanoid comparative genomics tool [28]. Squares represent the presence and absence of individual genes associated with each locus based on PyParanoid presence-absence data.

To assess the conservation of previously described insecticidal genes within the *Pseudomonas* genus, we conducted a comprehensive literature review and identified 302 genes previously implicated in insecticidal activity, virulence, or biocontrol against eukaryotic or prokaryotic organisms including insects, bacteria, and fungi. Using a comparative genomics database (28), we mapped the presence and absence of insecticidal and biocontrol genes onto a phylogenetic tree and visualized the distribution of these genes across different *Pseudomonas* groups (Fig. 1; Fig. S1). This analysis revealed variability in insecticidal and biocontrol gene presence between phylogenetic groups of *Pseudomonas*. Specifically, strains from the closely related subgroups *P. protegens* and *P. chlororaphis* have the highest number of insecticidal and biocontrol genes, with approximately 240 and 160 genes present, respectively, from a total database of 302 query genes (Fig. 1; Fig. S1; Table 1; Table S1). As a result, we further investigated the distribution of insecticidal genes within the *P. protegens* and *P. chlororaphis* subgroups (Fig. S2). Notably, the *fit* insect toxin gene cluster (*fitABCDEFGH*) and genes encoding the novel protein IPD072Aa were found to be uniquely associated with *P. protegens* and *P. chlororaphis*. IPD072Aa protects against Coleopteran pests when expressed in genetically modified plants (29) and was only found to be present in *P*. sp. WCS374, *P. protegens* Pf5*,* and *P. chlororaphis* O6 (Fig. 1; Fig. S2). These results suggest that some insecticidal mechanisms may be unique to subgroups of *Pseudomonas*.

**TABLE 1:** *Pseudomonas* strains used for the experiments in this study

Strain	Group	Subgroup	Number insecticidal or biocontrol genes^*a*^	Oral insecticidal activity^*b*^	Pathogenic to plants
DSM21245	*P. putida*	*P. putida*	75	This study	No
cit7	*P. syringae*	*P. syringae*	75	This study	No
B728a	*P. syringae*	*P. syringae*	103	This study	Yes
O6	*P. fluorescens*	*P. chlororaphis*	163	This study	No
CMR5c	*P. fluorescens*	*P. protegens*	213	This study	No
CMR12a	*P. fluorescens*	*P. protegens*	147	This study	No
Pf5	*P. fluorescens*	*P. protegens*	240	This study	No
CHA0	*P. fluorescens*	*P. protegens*	229	This study	No
WCS365	*P. fluorescens*	*P. brassicacearum*	143	This study	No
N2C3	*P. fluorescens*	*P. brassicacearum*	159	This study	Yes
N2E2	*P. fluorescens*	*P. brassicacearum*	155	This study	No
PAO1	*P. aeruginosa*	*P. aeruginosa*	137	This study	Yes
PA14	*P. aeruginosa*	*P. aeruginosa*	139	This study	Yes

^*a*^ Number of insecticidal and biocontrol genes as identified in the literature and shown in Fig. 1.

^*b*^ Oral insecticidal activity in *D. melanogaster* was tested in this study for all strains.

We also observed *Pseudomonas* subgroup-specific distributions of additional previously described insecticidal and biocontrol genes. The phenazine biosynthesis cluster, responsible for the production of the antimicrobial compound Phenazine-1-carboxylic acid (PCA) was conserved in *P. aeruginosa* and *P. chlororaphis* but was absent in all other *Pseudomonas* groups including *P. protegens* (Fig. 1). Conversely, genes encoding 2,4-Diacetylphloroglucinol (DAPG) biosynthetic enzymes, a known antifungal biocontrol molecule, were exclusive to *P. fluorescens* and *P. protegens* (Fig. 1; Fig. S2). Interestingly, genes encoding the high-molecular-weight Tc toxin (Tcc) were distributed across the *P. fluorescens* group but absent in both *P. protegens* and *P. chlororaphis*. These findings suggest that insecticidal and biocontrol genes are differentially distributed across the *P. fluorescens* group, with most genes from Table 1 conserved in the *P. protegens* and *P. chlororaphis* subgroups. However, functional testing is required to elucidate the role of these gene distributions in insect-killing activity.

### Natural variation of *Pseudomonas* insecticidal activity in *Drosophila* spp.

**Fig 2:**
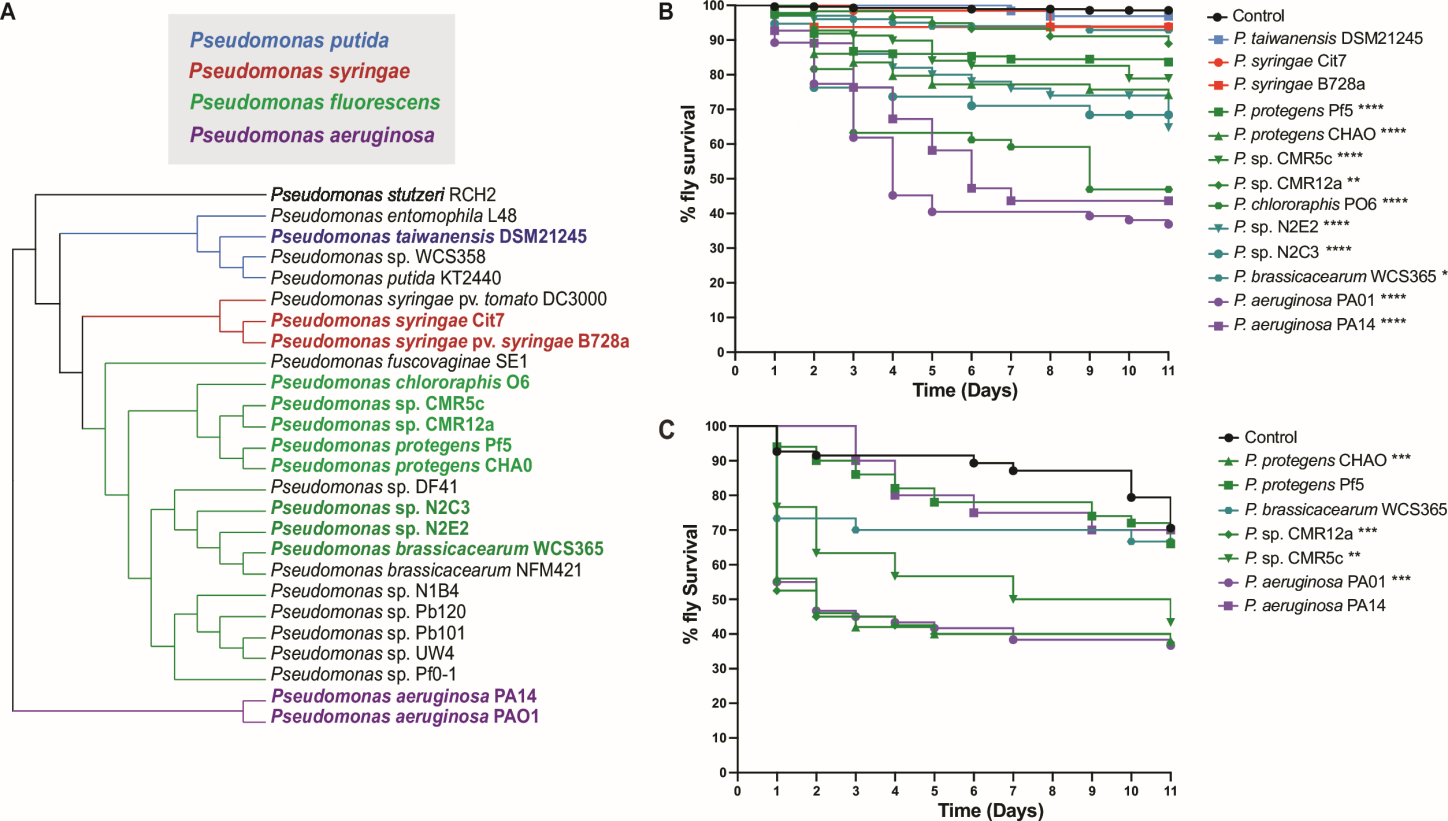
*Drosophila melanogaster* survival after oral infection with diverse *Pseudomonas* strains. (**A**) Phylogenetic tree of *Pseudomonas* strains tested in the fly survival assay. Colors indicate different *Pseudomonas* species: *P. putida* (blue), *P. syringae* (red), *P. fluorescens* (green), and *P. aeruginosa* (purple). (**B**) Kaplan-Meier (KM) survival curves of *D. melanogaster* Oregon-R flies following oral infection with *Pseudomonas* spp. strains Pf-5, CHA0, CMR5c, CMR12a, cit7, WCS365, N2E2, N2C3, DSM21245, B728a, PO6, PA14, PA01 (OD600 = 100), or with 5% sucrose solution (mock control). For *D. melanogaster,* each treatment group included 15–20 flies per replicate, and data were pooled from three independent experiments. (**C**) Kaplan-Meier (KM) survival curves of *D. suzukii* flies following oral infection with *Pseudomonas* strain Pf5, CHA0, CMR5c, CMR12a, WCS365, PA14 and PA01 (OD600 = 100), or control 5% sucrose solution. Each replicate included 5–10 flies. Statistics (for panels B and C): Pairwise Mantel–Cox (log-rank) tests were performed between infection conditions. Significance is shown as *P* ≤ 0.05 (*)*, P ≤* 0.01 (**)*, P ≤* 0.001 (***), *P* ≤ 0.0001 (****). Blank squares represent non-significant comparisons (*P* > 0.05); gray squares indicate comparisons that were not applicable. Corresponding number of subjects at risk, hazard ratios, and multiple comparisons are provided in Fig. S3.

To determine which *Pseudomonas* strains exhibit insecticidal activity against *D. melanogaster* (and the closely related agricultural pest *D. suzukii),* we orally infected flies with representative strains from the different *Pseudomonas* subgroups and monitored their survival over 11 days (Fig. 2; Fig. S3A through D). Kaplan-Meier survival analysis, including log-rank tests (relative to controls) and pairwise comparisons, revealed a broad range of insecticidal activity among *Pseudomonas* strains. *P. putida* (DSM21245) and *P. syringae* (cit7 and B728a) strains were not significantly different from controls, with 95%–100% insects surviving to the end of the experiment. In contrast*, Pseudomonas brassicacearum* WCS365 and *Pseudomonas* sp. CMR12a resulted in ~90% *D*. *melanogaster* survival but were significantly more pathogenic (*P* < 0.05 and *P* < 0.01, respectively). A second group of strains, including CMR5c, N2E2, N2C3, Pf5, and CHA0, decreased *D. melanogaster* survival to 90%–60% survival by day 11 (*P* < 0.0001). The most virulent strains, *P. aeruginosa* (PA14, PAO1) and *P. chlororaphis* (PO6), reduced *D. melanogaster* survival to ≤50% (*P* = 0.001) (Fig. 2A and B). These findings highlight significant natural variation in oral insecticidal activity within the *Pseudomonas* genus against *D. melanogaster*.

To quantitatively differentiate the killing profiles of *Pseudomonas* strains against *D. melanogaster*, we applied a Cox proportional hazards model to estimate hazard ratios (HRs) for each strain (Fig. S3E). HR values confirmed the trends observed in survival curves, separating strains into low (HR 1–5, *P* < 0.01), intermediate (HR 10–20, *P* < 0.001), and high (HR > 20, *P* < 0.001) killing groups. These results suggest that *Pseudomonas* strains exhibit discrete killing profiles against *D. melanogaster*, potentially governed by shared genetic and molecular mechanisms.

*D. suzukii*, or the spotted wing *Drosophila*, is a major agricultural pest capable of laying eggs in ripening fruits, causing significant economic losses (30, 31). Given the strong insecticidal effects of certain *Pseudomonas* strains on *D. melanogaster*, we tested whether these strains also affect *D. suzukii*. Adult flies were orally infected with selected *Pseudomonas fluorescens* species complex strains (CHA0, Pf5, CMR12a, CMR5c, WCS365) and *Pseudomonas aeruginosa* strains (PA14, PAO1), and survival was monitored over 11 days (Fig. 2C). Control flies (5% sucrose) showed minimal mortality, maintaining >90% survival until day 10. Among the tested strains, CMR5c, CMR12a, CHA0, and PAO1 were highly virulent, reducing survival to <50% by day 11 (log-rank test, *P* ≤ 0.001). In contrast, Pf5, WCS365, and PA14 caused moderate mortality, with 70%–80% survival by day 11 and non-significant differences compared to the control (Fig. 2C; Fig. S3F). These results indicate that while there is overlap in the killing profiles against these closely related fly species, they have distinct susceptibilities to some bacterial strains, in particular, *P. aeruginosa* species.

To robustly test whether insecticidal activity against *D. melanogaster* would be predictive of killing *D. suzukii,* we performed a linear regression to test the association between the survival of each species. We found a weak, and not statistically significant, positive linear relationship between the hazard ratios of the strains tested in both insect models, as indicated by a Pearson correlation coefficient of 0.2557 (*P* = 0.580) (Fig. S3G). A Spearman correlation analysis yielded a coefficient of 0.3214 (*P* = 0.498). This indicates that insecticidal activity against one species may not be broadly predictive, against even members of the same insect genus. Based on these findings, we cannot currently extrapolate the data from *D. melanogaster* to *D. suzukii,* further emphasizing the need for novel approaches to identify insecticidal genes and traits. Moving forward, we decided to explore exclusively insecticidal activity in *D. melanogaster.*

### Cox proportional hazard modeling and random forest analysis predict entomopathogenic genes

**Fig 3:**
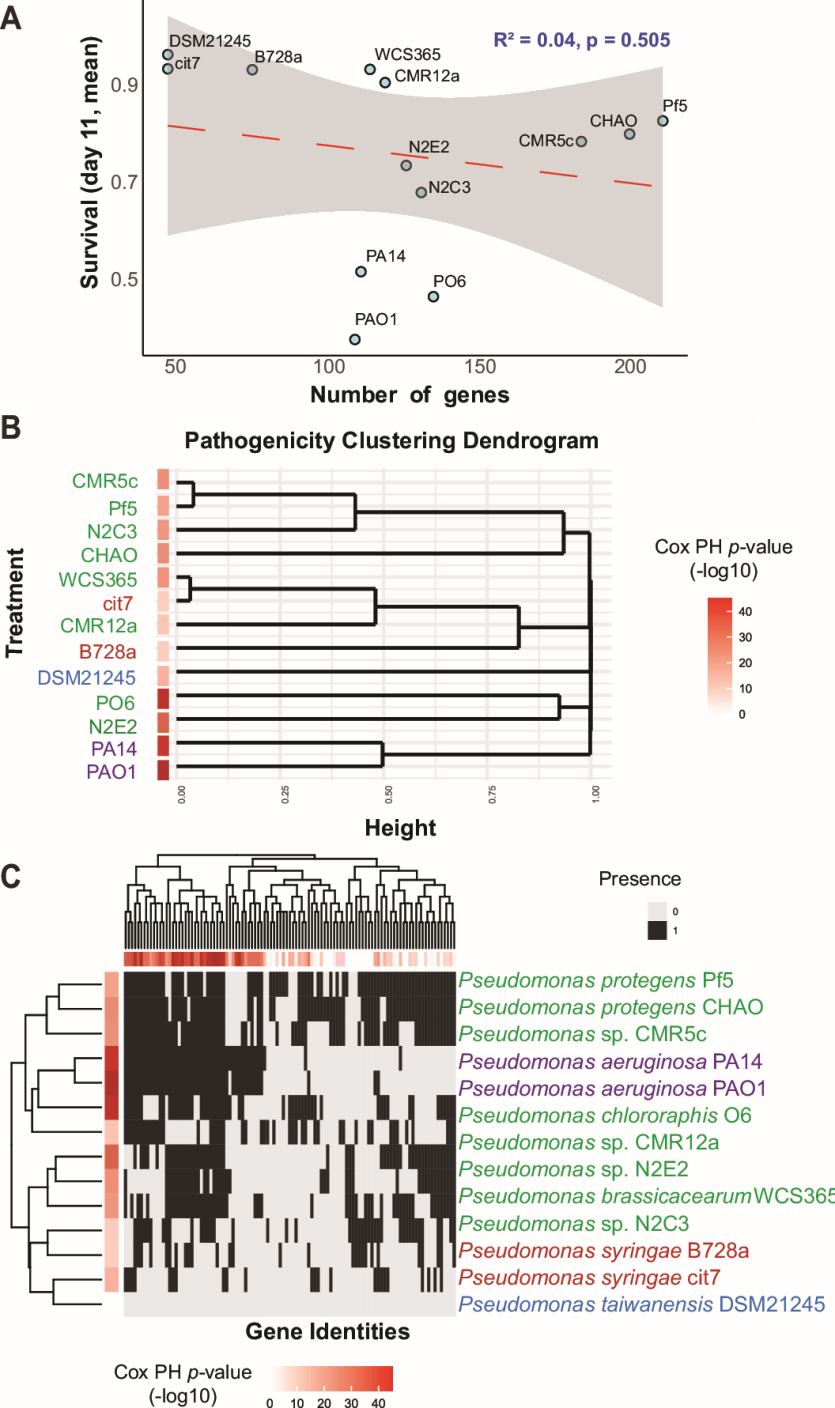
The presence of insecticidal and biocontrol genes, but not phylogeny, predicts insecticidal activity within the genus *Pseudomonas*. (**A**) Linear regression on *D. melanogaster* survival on day 11 and the number of predicted insecticidal genes in the given strain. The shaded region represents the 95% confidence interval for the linear regression (red line). (**B**) Hierarchical clustering of bacterial strains based on pairwise long-rank distance (1p) derived from Kaplan Meier survival curves (UPGMA, *k* = 5). (**C**) Hierarchical clustering between genes and strains based on binary gene presence/absence (Euclidean distance, complete linkage, *k* = 5). The top dendrogram shows genes clustered by their similarity across strains, while the side dendrogram shows strains clustered based on the similarity of their gene profiles. The horizontal red bars above the heatmap represent the *P-*values for how significantly each gene correlates with killing ability. The vertical red bars on the left side of the heatmap represent each strain’s proportional hazard (i.e., killing ability).

We conducted a correlation analysis to explore the relationship between the insect-killing activity of bacterial strains and their genetic composition. Specifically, we examined the correlation between the percentage survival of *D. melanogaster* by day 11 and the number of insecticidal and biocontrol genes present in the strains we inoculated (Table 1; Table S1). The analysis revealed a weak negative correlation, as shown in Fig. 3A, indicating that strains with more predicted insecticidal genes were associated with lower fly survival rates, suggesting increased killing activity. The *R*-squared value of 0.04 (*P* = 0.505) indicates that the total number of these genes does not explain the observed insecticidal activity (Fig. 3A). Consistent with host-specific virulence mechanisms, this suggests that the absolute number of biocontrol and insecticidal genes does not predict the insecticidal activity of *Pseudomonas* strains against *D. melanogaster*.

*Pseudomonas* virulence genes can be horizontally transferred over short evolutionary distances (32, 33). At the same time, other traits correlate with phylogeny (34, 35), we hypothesized that insecticidal activity may be influenced by both phylogeny and gene presence/absence patterns, but to different extents. From Fig. 1B, we observed that *P. aeruginosa* and *P. protegens* strains exhibited higher insecticidal activity than other *Pseudomonas* groups. To explore whether phylogeny explains this pattern, we clustered strains based on their killing profile (Fig. 3B) and compared them to phylogeny. To test whether insecticidal activity and the presence/absence of known insecticidal genes correlate with phylogeny, we constructed a dendrogram based on gene presence/absence (Fig. 3C). We quantified these relationships using the Adjusted Rand Index (ARI), which measures clustering similarity while accounting for random agreement (−1 = no agreement, 1 = perfect agreement). Pathogenicity showed a weak correlation with phylogeny (ARI = 0.1954, *P* = 0.082), suggesting phylogeny alone does not explain killing profiles. However, gene presence/absence clustering showed a slightly better correlation with pathogenicity (ARI = 0.287, *P* = 0.029), indicating that certain genes better predict insecticidal activity. The strongest correlation was between gene presence/absence and phylogeny (ARI = 0.6325, *P* < 0.001), suggesting that while phylogeny shapes the distribution of insecticidal genes, specific genes may play a key role in pathogenicity.

**Fig 4:**
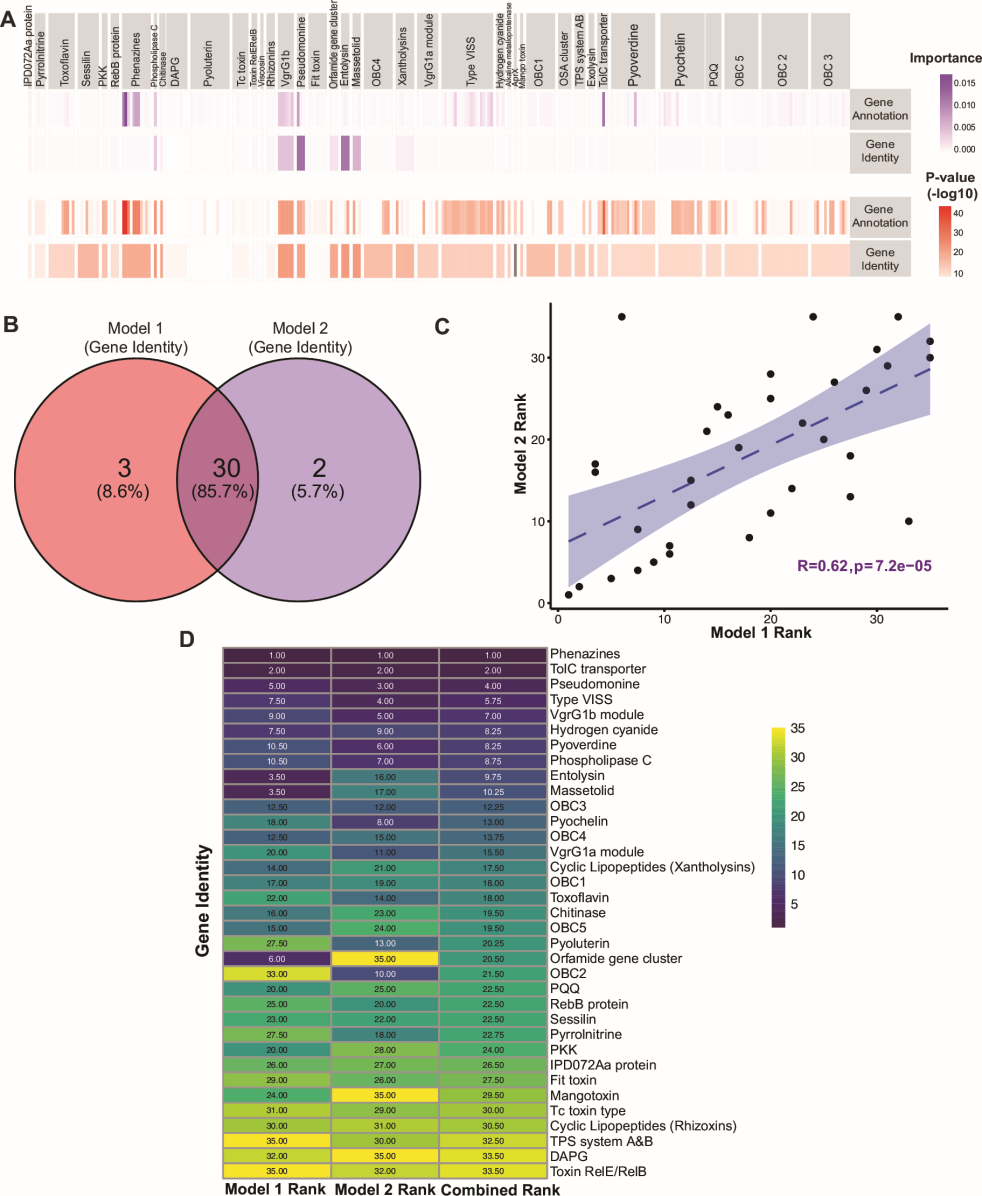
A subset of insecticidal and biocontrol genes predicts virulence against *D. melanogaster*. (**A**) Statistical models: Cox proportional hazard modeling and random forest analysis. The purple boxes on the top horizontal row show the individual gene identity risk Importance-value using the random forest analysis. The red boxes on the top horizontal row show the individual gene identity risk *P*-value using the Cox proportional hazard on individual genes. (**B**) Venn diagram of gene identities for each model. Central overlap: shared genes. Unique segments: genes unique to each model (red segment for Model 1, purple segment for Model 2). (**C**) Positive correlation between gene identity rankings in Model 1 (*y*-axis) and Model 2 (*x*-axis) (*P* < 0.05). (**C**) Venn diagram of gene identities for each model. Central overlap: shared genes. Unique segments: genes unique to each model (red segment for Model 1, purple segment for Model 2). (**D**) Heatmap of the ranking of gene identities for each model. The *x*-axis shows the gene identities and the *y*-axis the different rankings. Model 1 ranking is based on *P*-value and coefficient, Model 2 ranking is based on the Importance-value > 1, and average ranking is a combination of both rankings.

We asked whether certain genes are better predictors of the killing of *D. melanogaster*. To address this, we employed two complementary strategies. In Model 1, we used a Cox proportional hazards model to assess each genetic predictor individually, iterating through genes from our comprehensive database. This method fitted separate Cox models for each predictor against entomopathogenicity (Fig. 4A; Fig. S4A). Genes strongly associated with increased mortality (high hazard ratios and low *P-*values) were visualized using a volcano plot (Fig. S4B). Using this model, we identified 33 distinct gene identities significantly correlated with entomopathogenicity (Fig. 4B). To address potential false positives that may arise due to the correlation of insecticidal activity and strain phylogeny, we employed a dual-method approach that combined the Cox PH model with a Random Forest model (Model 2). This method evaluated all genes collectively, considering co-occurrence patterns and interdependencies. We ranked gene identities based on *P*-value and coefficient (Model 1) and importance value (Model 2), finding a strong positive correlation between the rankings, indicating that both models prioritized gene identities in a similar order (Fig. 4C). The genes identified through this model (Fig. 4A, purple panel) were largely a subset of those identified by the Cox model (Model 1). Model 2 identified 32 significant gene identities, including 30 overlapping with Model 1 and 2 unique genes to Model 2 (Fig. 4B). The high degree of overlap from both models, which use independent approaches, suggests the 30 genes identities identified by both models are strong candidates for genes important for insecticidal activity against *D. melanogaster.*

### Modeling predicts genes necessary for insecticidal activity in *Drosophila melanogaster*

**Fig 5:**
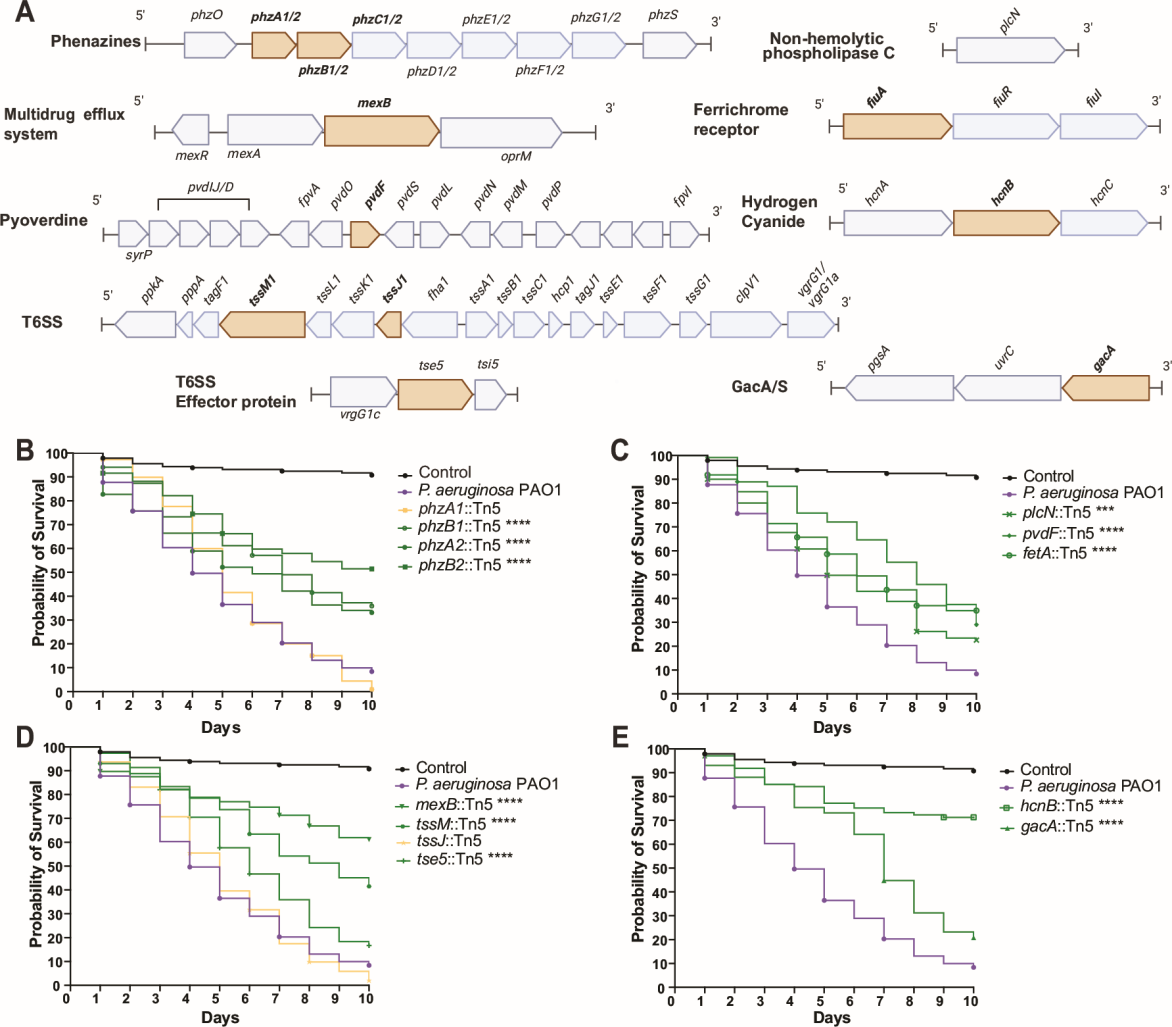
Modeling approaches identified *Pseudomonas* genes that are necessary for insecticidal activity in *Drosophila*. Genes predicted by both models (Fig. 4) were validated using *P. aeruginosa* PA01 (H103) and PAO1 transposon mutants. (**A**) Schematic representation of the seven distinct genes and operons selected from the models; the gene in tan indicates the location of the transposon insertion. (**B**) Kaplan-Meier (KM) survival curves of *D. melanogaster* Oregon-R flies following oral infection with *P. aeruginosa* PAO1 and transposon insertion mutants in genes encoding phenazine biosynthesis (*phz1 and phz2*); (**C**) PlcN, ferrichrome receptor A (*fiuA*), and pyoverdine biosynthesis; (**D**) TolC transporter (*mexB*), and the Type VII secretion system (*tssM* and *tssJ*), including module VgrG1b (*tse5*); (**E**) GacA/S two-component system and hydrogen cyanide.

To determine if testing a limited number of strains could predict genes important for killing a new host, we tested mutants in genes predicted by our modeling approach (Fig. 4). To validate the model, we made use of a previously described *P. aeruginosa* PAO1 transposon mutant library (36). We chose 7 of the 30 genetic clusters to test through ranking based on the *P-*value and coefficient (hazard ratio), (Model 1) and gene normalized importance from the Random Forest algorithm (Model 2). We integrated findings from both models to identify high-ranking genes (Fig. 4D). These genes included those involved in the production of phenazines, cyclic lipopeptides, and type VI secretion systems. We selected seven functionally distinct genes or operons or individual genes for testing, prioritizing those involved in different processes, including genes previously reported insecticidal or genes for biocontrol but not insecticidal activity (Table 2). These genes include phenazines, MexAB-OprM efflux pump, phospholipase C, the Type VII secretion system, pyoverdine, ferrichrome receptor A (fetA-like protein) and hydrogen cyanide. Additionally we included the GacA/S two-component system as a positive control for virulence, given its well-established role as a master regulator of multiple virulence pathways in *Pseudomonas* (Fig. 5A). Several top-ranked genes (e.g., hydrogen cyanide genes) have been independently reported as insecticidal in *D. melanogaster* (37), providing an internal validation that our strain-level model can rediscover some virulence factors. Conversely, candidates such as MexAB-OprM and several siderophore-related (e.g., pyoverdine and pyochelin) loci have not previously been implicated in virulence in *D. melanogaster,* illustrating the framework’s capacity to identify virulence genes in a new host.

**TABLE 2:** Mutant genotypes in *P. aeruginosa* PAO1 used in this study

Strain	Gene input	Transposon insertion in PAO1	Operon	Predicted defective in production of	PA ORF
*Pseudomonas aeruginosa* PAO1	*phzA1*	*phzA1*	*phz I/R/A/B/C/D/E/F/G/H/O*	Phenazines Operon 1	PA4210
	*phzB1*	*phzB1*		Phenazines Operon 1	PA4211
	*phzA2*	*phzA2*		Phenazines Operon 2	PA1899
	*phzB2*	*phzB2*		Phenazines Operon 2	PA1900
	*mexB*	*mexB*	*mexR/A/B* and *oprM*	RND multidrug efflux	PA0426
	*pvdF*	*pvdF*	*pvdS/G/L/H/I/J/D/E/F/O/N/M/P/T/R/A/Q* *PA2411/PA2412* *fpvF/E/D/C/K/J/H/G/A/R/I*	Pyoverdine	PA2396
	*fetA*	*fiuA*	*fiuA/R/I*	Ferrichrome receptor A	PA0470
	*tssM*	*tssM*	*tagQ/R/S/T/F/H ppkA pppA tssM/L/K/J/A/B/C/E/F/G hcp*	T6SS	PA0077
	*tssJ*	*tssJ*		T6SS	PA0080
	*tse5*	*rhsA*	*Effector protein ts5*	T6SS effector protein ts5	PA2684
	*plcN*	*plcN*	*plcN*	Phospholipase C	PA3319
	*hcnB*	*hcnB*	*hcn A/B/C*	Hcn A/B/C	PA2194
	*gacA*	*gacA*	*gacS/gacA*	GacA/S two-component system	PA2586

We found that of the 13 PAO1 mutants tested, 11 had significant loss in virulence in *D. melanogaster*. These include transposon insertions in *phzB1, phzA2, phzB2, mexB, pvdF, fiuA, tssM, tse5, plcN, hcnB,* and *gacA* (Fig. 5; Fig. S5). Disruption of phenazine production (*phzA2, phzB2,* and *phzB1*) significantly reduced virulence though complete survival restoration may be hindered by redundancy between the two phenazine operons (38) (Fig. 5B). Mutants in *mexB* (MexAB-OprM Efflux Pump) and *plcN* phospholipase C and *pvdF* and *fiuA* (siderophore biosynthesis and iron acquisition) also demonstrated markedly reduced virulence (Fig. 5C and D). Loss of *tssM* and *tse5* impaired T6SS function, with the *tssM::*Tn5 mutant showing insecticidal activity reduction (Fig. 5D). The *hcnB* mutant showed the highest virulence attenuation, underscoring the role of hydrogen cyanide in infection (Fig. 5E). Surprisingly, the *gacA* mutant exhibited only a slight virulence reduction, suggesting a subtler role for this regulator (Fig. 5E). Growth assays confirmed comparable growth rates among mutants and wild type when growing in minimal media supplemented with fly extract, ruling out generalized growth defects as the cause of virulence loss (Fig. S6). These findings show that our models successfully identified genetic pathways required for the *Pseudomonas* pathogenicity of flies, indicating that comparative genomics, coupled with testing a small number of strains successfully identified virulence factors in a new model.

To quantitatively assess how well our models predict experimental survival outcomes, we compared model rankings with survival significance using Fisher’s method to aggregate *P*-values from replicate Kaplan–Meier assays. Fisher’s method provided a single, robust significance score for each gene. We tested alternative metrics (e.g., composite scores integrating hazard ratios and confidence intervals) and found similar correlation trends. This analysis revealed that Model 1 was the strongest predictor of survival (Spearman *R* = −0.86, *P* = 0.0068), with the combined ranking performing similarly (*R* = −0.83, *P* = 0.011), while Model 2 (*R* = −0.62, *P* = 0.11) alone showed lower predictive alignment (Fig. S7). Collectively, this finding shows that we were effectively able to use existing literature on the genetics of insecticidal mechanisms, and testing a limited number of bacterial strains, to determine which known insecticidal genes were effective on a new host.

## DISCUSSION

In this manuscript, we explored the possibility of combining existing literature with functional tests from a limited number of strains to identify which previously described insecticidal genes are effective in a new host. We evaluated the genetic basis of insecticidal activity in target *Pseudomonas* strains against *Drosophila melanogaster* and, using robust statistical models, predicted which genes were crucial for killing activity against *D. melanogaster*. By integrating Cox proportional hazards modeling with Random Forest analysis, we developed a system capable of distinguishing key virulence and entomopathogenicity genetic determinants based on the killing profile of individual strains. This approach successfully identified both previously described and undescribed genes that are necessary for virulence on *D. melanogaster.* This indicates that our approach can advance our understanding of microbial entomopathogenicity.

The Cox proportional hazards model identified a broad set of genes significantly associated with reduced survival in *Drosophila melanogaster*, while Random Forest analysis highlighted a subset of these genes under stricter criteria, thereby minimizing the likelihood of false positives. The intersection of these models revealed 30 high-confidence gene candidates, including those involved in phenazine production, efflux pumps, siderophores, T6SS, and hydrogen cyanide synthesis. These findings emphasize the importance of using diverse statistical methods to enhance predictive accuracy.

The genes identified by both models underscore the complexity of insecticidal activity, where multiple genetic pathways converge to mediate virulence. For example, phenazine operons, previously associated with antimicrobial activity (38–40), emerged as significant determinants of insect mortality, suggesting a dual role in bacterial fitness and pathogenicity. Similarly, the MexAB-OprM efflux pump and some siderophores were shown to be critical for killing efficiency, likely by helping bacteria evade the insect immune barriers. These results highlight how statistical modeling can uncover genes with roles in virulence that might be overlooked in traditional single-gene studies.

We found a weak correlation (*R*² = 0.04) between the total number of insecticidal genes identified through a literature search and observed virulence, indicating that the presence of a larger number of putative insecticidal genes does not necessarily translate into higher virulence. This may be due to functional redundancy, host-specificity, or the possibility that not all insecticidal mechanisms contribute equally to insect killing. This suggests that insecticidal activity against one species may not be broadly predictive, even within members of the same insect genus. Instead, our models demonstrate that specific genes better predict insecticidal profiles. The models developed in this study have broad applications beyond the insecticidal activity of the genus *Pseudomonas*. By prioritizing genes based on their statistical and functional significance, our approach can be adapted to other microbial systems to identify key determinants of pathogenicity or biocontrol potential. These insights also inform the design of biocontrol agents, enabling the selection of strains enriched with high-impact genes while minimizing unintended effects on non-target organisms. For example, our results reveal that different *Pseudomonas* strains exhibit distinct virulence patterns in *D. melanogaster* and *D. suzukii,* suggesting host specificity of virulence mechanisms. The modeling framework presented here may be applicable for testing such hypotheses in future research.

Our gene database was compiled from published studies, many of which focused on genes within the genus *Pseudomonas* that have activity against Lepidopteran and Coleopteran pests or the model organism *Drosophila melanogaster*, reflecting the research bias in the field. While this broad approach maximizes gene coverage within *Pseudomonas*, it introduces potential limitations because virulence mechanisms can be highly host-specific and may not transfer across insect orders. Furthermore, these mechanisms may not be present in other bacterial taxa. Additionally, our current model treats genes as independent predictors rather than grouping them into functional clusters (e.g., the *Fit* toxin operon). This design choice reflects our goal of developing a rapid, agnostic screening framework that does not rely on extensive manual curation or prior assumptions. However, future refinements could incorporate curated gene clusters and host-specific data sets to improve biological interpretability and predictive power, particularly for applications across multiple insect hosts.

By using a combined approach, this study provides information on which genes to prioritize when screening insecticidal strains from rhizosphere communities. Using the results from our statistical model, it is possible to analyze new soil or environmental bacterial isolates to identify genes associated with the desired insecticidal phenotype. While the identified genes provide a strong starting point for understanding insecticidal phenotypes, their effectiveness against entirely new insect targets remains uncertain. Instead, the strength of this approach lies in its ability to guide the discovery of novel insecticidal traits tailored to agricultural applications. Future studies could explore whether the statistical models and identified genes can help predict insecticidal activity in diverse ecological contexts. This would involve validating the approach in systems with varying host-pathogen interactions and adapting it for targeted screening efforts to meet specific agricultural needs. Collectively, this study demonstrates the use of statistical models in identifying key genetic drivers of insecticidal activity, offering a framework for understanding microbial pathogenicity and its applications in biocontrol.

## MATERIALS AND METHODS

### Bacterial strains and growth conditions

All the *Pseudomonas* strains and mutants used in this study are summarized in Tables 1 and 2. Strains were streaked on LB agar and incubated at 28°C or 37° (*P. aeruginosa* only) for 24 h. Overnight cultures were made from single colonies, transferred to 100 mL of 3% NaCl LB, grown at 28°C or 37°C (*P. aeruginosa* only), and shaken at 180 rpm. PAO1 transposon insertion mutants were obtained from the PAO1 transposon insertion library (36). Wild-type PAO1 and the transposon insertion mutants used in this study were cultured in LB. The initial screen (Fig. 1B) was conducted using *Pseudomonas aeruginosa* PAO1 (H103), and while the reported parental strain of the transposon mutant library, *Pseudomonas aeruginosa* PAO1 (MPAO1), displayed slightly reduced killing activity compared to H103 (Fig. S8A), as an additional control, we chose to test neutral mutants from the library for comparison. Our analysis showed no significant difference in killing activity between the neutral mutants and the H103 strain (Fig. S8B) suggesting a loss of virulence with the designated “parental” strain in our library.

### Bacteria preparation for *D. melanogaster* and *D. suzukii* oral infections

Bacteria for infecting flies were prepared as described (25). Freezer stocks were streaked onto LB-milk plates and incubated the plate at 28°C for 2 days. Colonies were selected and inoculated into 100 mL cultures in 3% NaCl LB broth and shakinge at 180 rpm for at least 20 h at 28°C. Subsequently, cells were pelleted by centrifugation at 2,500 × *g* for 30 min at 4°C. After pelleting, the supernatant was centrifuged again to confirm the removal of most bacteria. The culture was then concentrated by resuspending the pellet in 1 mL of supernatant. The OD_600_ was measured against a 3% NaCl LB broth blank using 1 mL of liquid per cuvette, using 1 in 100 dilutions of the bacterial concentrates.

### *D. melanogaster* and *D. suzukii* growth conditions

The *D. melanogaster* Oregon R colony was grown for 16 h of light and 8 h of dark at 24.5°C and 22.5°C, respectively, with 50% humidity. Flies for the initial screen, Fig. 2A, were reared in freshly made Lewis medium. Flies for validation experiments (Fig. 5) were reared in Bloomington media. *D. suzukii* was reared in milk bottles containing a standard potato flake diet (Ward’s Instant Drosophila Medium) (41). These bottles were incubated under a 16 h light:8 h dark at 24.5°C and 22.5°C.

### *D. melanogaster* and *D. suzukii* oral infection assay

Inoculum preparation, bacteria were concentrated to an OD_600_ of 100 in 5% sterile sucrose. To prepare flies, 2-5 days-old adult flies were collected and starved for at least 2 h before the infection. Using forceps, filter paper discs were placed on top of the fly food, and 220 µL of inoculum was pipetted directly onto the filter paper. Once all vials were inoculated, we waited for 10 min for the inoculum to absorb into the filter paper before transferring flies into the infection tubes. Tubes were then incubated at 25°C, and fly deaths within 2 h were recorded, which were considered infection-independent deaths. The oral infection process was allowed to proceed for 24 h, during which fly deaths were recorded. Following this initial 24 h period, the flies were transferred to a fresh food source, and subsequent fly deaths were recorded daily over 10 days. Fly oral infection was repeated 3 times for each bacterial strain with 20 flies per replicate.

### Clustering and ARI comparisons analysis

We compared three structures: (i) killing profile clusters (derived from hierarchical clustering of log-rank distance matrices; Fig. 3B), (ii) gene presence/absence clusters (hierarchical clustering of binary gene matrices; Fig. 3C), and (iii) phylogeny-defined groups (from the phylogenetic tree described in Fig. 1B). ARI values were computed using *mclust::adjustedRandIndex* function. Clusters were defined by cutting dendrograms at *k* = 5; analyses with *k* = 3–5 gave similar rankings, supporting robustness. Statistical significance of ARIs was assessed vis 1,000 permutations of cluster labels.

### Statistical curve analysis

Survival data in the *D. melanogaster* oral infection survival assays were analyzed using Prism 9 software using the Log-Rank test of the Survival package and the one-way ANOVA test. The hazard ratio for each of the strains compared to the control group was calculated using the Cox proportional hazard model in R (2022.2.0.443).

### Identification of insecticidal and biocontrol genes from the literature

A list of bacterial genes reported to be involved in insecticidal activity toward different insects and the biocontrol of microbial pests was compiled. For each of the genes, amino acid sequences were collected from the NCBI gene bank and the *Pseudomonas* Genome Database. A database of 302 genes, ranging from single genes to complete operons, was included in the analysis (Table S1). These annotations were grouped into 44 gene identities (functional categories) that formed the basis of our modeling approach, as decisions for mutant validation were made at the level of gene identities rather than individual annotations. Genes associated with insect killing were sourced from studies spanning multiple insect hosts, including Lepidoptera, Diptera, and Coleoptera. While this provided a broad database of putative virulence factors, we recognize that such heterogeneity may bias predictions when applying the model to a specific host.

### Bioinformatic analysis

*Phylogenetic tree construction:* A phylogenetic tree of bacterial strains was constructed using protein sequences extracted from each genome. The standalone OMA (Orthologous Matrix) algorithm (42) was employed with recommended parameter settings for the analysis. OMA groups were filtered using the filter_groups.py script (43), applying a minimum species coverage threshold of 10 out of 27 genomes. Orthologous groups were aligned with MAFFT (v7.526), allowing a maximum of 1,000 iterative refinements. The resulting alignments were concatenated using the concat_alignments.py script (43). Finally, a phylogenetic tree was generated using FastTree (v2.1.11).

*Plotting presence and absence:* Using PyParanoid, a pipeline for the rapid identification of orthologous genes in a set of genomes (28) and FastTree 2a tool for inferring approximate-maximum-likelihood phylogenetic trees, a *Pseudomonas* species tree including representative strains from different clades was constructed (Fig. 1). Using Pyparanoid, we identified orthologous groups in the *Pseudomonas* strains and plotted the presence and absence of these insecticidal-associated genes against the species tree (Fig. S1).

### Statistical models

*Gene annotation data set preparation*: The data set consisted of bacterial gene metadata, including Gene ID, Gene Annotation, and Function. Gene ID represents the gene’s identity (e.g., *Fittoxin*), Gene Annotation describes genetic elements within the genome (e.g., *FitA, FitB*), and Function categorizes the gene based on literature into “Insecticidal,” “Biocontrol,” “Antimicrobial,” or various “Virulence factor” subtypes. The data set was preprocessed to remove: (1) Gene IDs and annotations are present in all or no treatments. (2) Gene IDs associated with only one treatment, as their effects could not be distinguished from strain-level effects. (3) Gene IDs with identical occurrence patterns, which were collapsed into a single concatenated ID.

*Cox Proportional Hazard Models (Model 1)*: To evaluate fly mortality, Cox Proportional Hazard (Cox PH) models were applied. Fly mortality risk for each bacterial treatment was assessed using the coxph function in R’s *survival* package. Hazard ratio plots were created using the gg-forest function from the survminer R package (44). Models included sex as a predictor, with females as the reference, and data were censored at 11 days post-exposure. Hazard ratios and BH-adjusted *P*-values for each gene were visualized in volcano plots (Fig. S4B) to identify genes significantly associated with fly mortality. For gene-level analyses, Cox PH models were used to assess the association between gene presence/absence and fly mortality across bacterial strains. To correct for multiple testing, the Benjamini-Hochberg (BH) adjustment was applied to *P*-values. Hazard ratios and adjusted *P*-values were used to prioritize candidate genes. Given the exploratory nature of this model, we did not test proportional hazards assumptions for each gene model. Instead, the Cox PH model served as a high-throughput screening step, followed by additional filtering via Random Forest importance and experimental validation. For the strain-level model, the proportional hazards assumption was tested using the *cox.zph* function. The full model (12 bacterial strains + control) showed a minor violation (*P* = 0.021), which was resolved by removing one outlier strain (Pf5, *P* = 0.12) (Fig. S9). Residual plots from the Schoenfeld test did not suggest strong deviations from the proportional hazards assumption (Fig. S9).

*Random forest model (Model 2):* A random forest model was constructed using the *randomForestSRC* R package to evaluate the collective effects of gene IDs on fly mortality. This approach complemented the individual Cox PH models by capturing potential interactions and co-occurrence patterns among genes, helping to mitigate false positives arising from univariate analysis. The model was trained with 500 trees, a default node size of 15, and the log-rank random split rule. Out-of-bag (OOB) error was estimated at 25.4%, indicating moderate predictive power when using gene presence alone. No cross-validation was applied due to the limited number of samples, and we emphasize that the goal of this model was not classification performance, but exploratory prioritization of candidate genes.

*Integrated ranking analysis*: To prioritize candidate insecticidal genes, we implemented a ranking-based integration of the two statistical models. For the Cox PH model (Model 1), genes were ranked based on a combination of effect size (coefficient) and statistical significance (BH-adjusted *P*-value). For the random forest model (Model 2), genes were ranked according to their normalized importance values. By averaging gene ranks across both models, we generated a composite gene ranking (Fig. 4D). Genes that consistently ranked within the top percentile in both models were classified as high-confidence insecticidal candidates and selected for downstream experimental validation. This integrative strategy was designed to reduce false positives and enhance the robustness of candidate selection by requiring the agreement of both models.

*Model ranking vs. survival prediction:* To determine which model best predicts fly survival outcomes (Fig. 5), we compared the gene rankings from Model 1, Model 2, and the combined model against experimental survival significance. For each gene, *P*-values from Kaplan–Meier survival analyses (across replicates) were aggregated using Fisher’s method, and significance was expressed as—log10(P-Fisher) (Fig. S7). We tested multiple survival metrics (e.g., composite scores combining *P*-value, effect size, and confidence interval width), all of which showed similar Spearman correlation trends. All analyses were conducted in R (4.2.1) using *dplyr*, *ggplot2*, *ggpmisc*, *ggpubr*, and *metap*.

## SUPPLEMENTAL MATERIAL

Supplemental material spectrum.03108-25-s0001.pdf Table S1; Fig. S1 to S9.

ASM does not own the copyrights to Supplemental Material that may be linked to, or accessed through, an article. The authors have granted ASM a non-exclusive, world-wide license to publish the Supplemental Material files. Please contact the corresponding author directly for reuse.
