# Supplementary material for: Predicting bacterial-mediated entomopathogenicity through comparative genomics and statistical modeling
Source: Microbiol Spectr. 2025 Nov 26;14(1):e03108-25. doi: 10.1128/spectrum.03108-25 (PMC12772284; doi:10.1128/spectrum.03108-25)
Supplement: Supplemental material — Table S1; Fig. S1 to S9. [file spectrum.03108-25-s0001.pdf]

Supplementary Information

Supplemental Tables and Figures

Table of insecticidal genes (references and insects)

Table S1. List of predicted insecticidal gene functions.

Identity	Gene Annotation	Source	Function
Hydrogen cyanide	*hcn A/B/C*	(1)	Insecticidal
Fit toxin	*fitA/B/C/D/E/F/G/H*	(2)	Insecticidal
IPD072Aa protein	*IPD072Aa*	(3)	Insecticidal
ß-Pore-Forming Toxin	*monolysin*	(4)	Insecticidal
Phospholipase C	*plcN*	(5)	Insecticidal
Alkaline metalloproteinase	*aprA*	(6)	Insecticidal
Two-component system	*gacS/gacA*	(5)	Insecticidal
Two-partner secretion toxin (Exolysin)	*ExlB/A*	(7)	Insecticidal
Orfamide gene cluster	*OfaA/B/C*	(8)	Insecticidal
Chitinase	*ChiC/D*	(9)	Insecticidal
Cyclic Lipopeptides (Rhizoxins)	*RzxB/C/D/E/F/G*	(9)	Biocontrol
Cyclic Lipopeptides (Xantholysins)	*ClpA/B/C/D/E/F/G/H*	(10)	Antimicrobial
2,4-diacetylphloroglucinol (DAPG)	*PhlA/B/C/D/E/F/G*	(5)	Antimicrobial
Phenazines	*phzI/R/A/B/C/D/E/F/G/H/O*<br>*glu*	(11)	Biocontrol
TPS System A and B	*tpsB1/A1/B2/A2/B3/A3/B4/A4*	(12)	Insecticidal
RebB protein	*rebB_1/reb_2/PPRCHA0_0184*	(13)	Insecticidal
OSA cluster	*PPRCHA0_4348-4354*	(12)	Insecticidal
O-antigenic polysaccharides (OBC1/OBC2/OBC3/OBC4/OBC 5)	*OBC1 (PFL_2023-2033)*<br>*OBC2 (PFL_3077-3094)*<br>*OBC3 (PPRCHA0_1948-1966)*<br>*OBC4 (PFL_5482-5496)*<br>*OBC5 (PFL_5091-5108)*	(12)	Insecticidal
Polyphosphate Kinase	*pap*	(12)	Insecticidal
Type VISS	*tagQ/R/S/T/F/H*<br>*ppkA*<br>*pppA*<br>*TssM/L/K/J/A/B/C/E/F/G*<br>*hcp*	(14)	Biocontrol
VgrG1a and 1b module	*vgrG1a* and *PPRCHA0_6003-6009*<br>*PPRCHAO_3010-3015*	(14)	Biocontrol
TolC transporter	*mexR/A/B* and *oprM*	(15)	Virulence factor
Pyoluterin	*pltL/M/R/A/B/C/D/E/G/Z/I/J/K/N/O /P/*	(16)	Antimicrobial
Pyrrolnitrine	*prnA/B/C/D*	(17)	Antimicrobial
Massetolid	*massA/B//C*	(18)	Antimicrobial
Sessillin	*sesA/B/C/T/R/D/B/C*	(8)	Antimicrobial
Metallopeptidase AprA	*aprX*	(5)	Insecticidal

Viscosin	*viscB/C*	(19)	Antimicrobial
PKK	*pkk1, pkk2A/B/C*	(20)	Virulence factor
Tc toxin	*tcaA1, tcaB1, tcdC1, tccC2_2, tccC2, Pfl01_0947*	(21)	Insecticidal
Toxoflavin	*toxH/G/M/R/E/C/B/D/A/F*	(22)	Antimicrobial
Pseudomonine	*pmsE/C/A/B*	(23)	Virulence factor (siderophores)
Pyochelin	*pchA/B/C/K/F/E/I/H/D/R* *fetA/B/C/D/E/F* and *PFL_3504*	(24)	Virulence factor (siderophores)
Pyoverdine	*pvdS/G/L/H/I/J/D/E/F/O/N/M/P/T/R/A/Q* *PA2411/PA2412* *fpvF/E/D/C/K/J/H/G/A/R/I*	(25)	Virulence factor (siderophores)
PQQ	*gcd* and *pqqH/I/J/K/M/E/D/C/B/A/F*	(26)	Antimicrobial
Toxin RelEB	*relE/B*	(27)	Virulence factor
Entolysin	*eltA/B/C*	(28)	Insecticidal
Mangotoxin	*mgoA*	(29)	Virulence factor (plants)

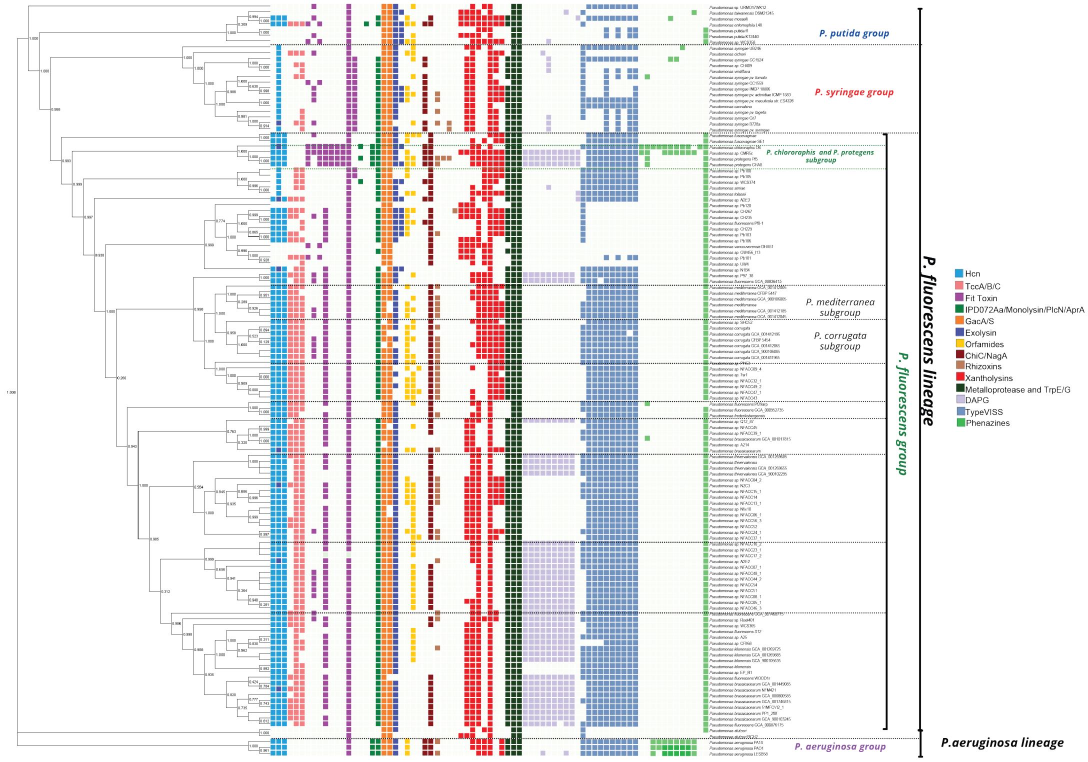

Figure S1: Distribution of insecticidal, biocontrol and antifungal genes within the *Pseudomonas genus.* The species tree was constructed using the PyParanoid comparative genomics tool. Squares represent the presence and absence of individual genes associated with each locus based on PyParanoid presence-absence data. Coloured squares represent the presence of a homologous gene, while absence is represented by white. The genus *Pseudomonas* is divided into 5 phylogenetic groups, *Pseudomonas aeruginosa, P. fluorescens*, *Pseudomonas putida and Pseudomonas syringae.* Within the *P. fluorescens* group, we have other subgroups such as *P. chlororaphis* and *P. protegens.*

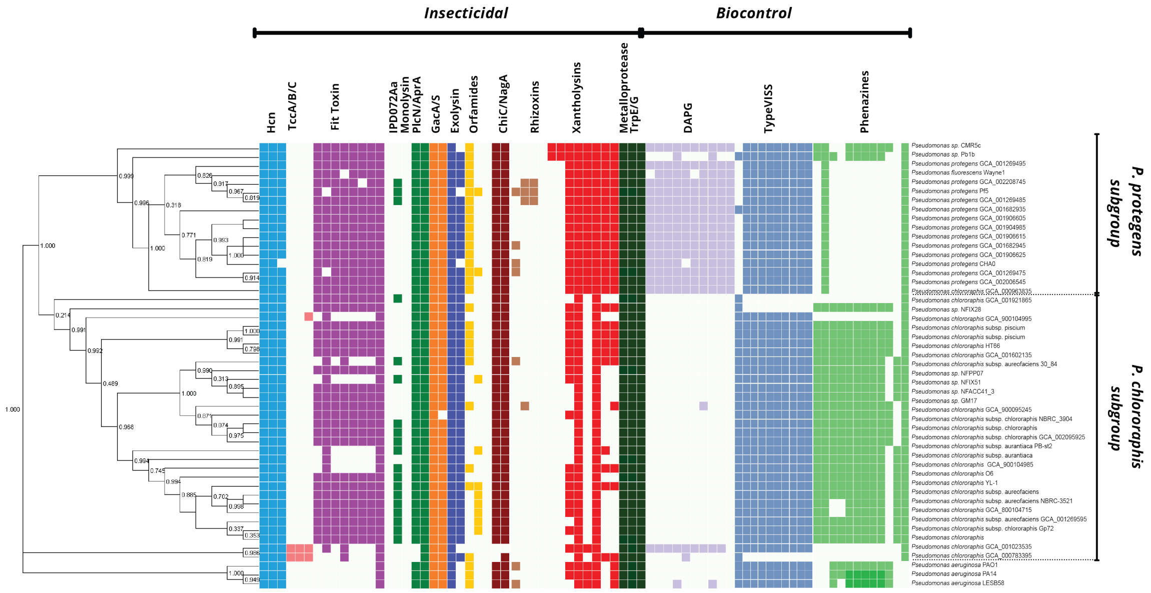

Fig S2. Distribution of insecticidal, biocontrol and antifungal genes within the *Pseudomonas protegens* and *chlororaphis subgroup*. The species tree was constructed using the PyParanoid comparative genomics tool. Squares represent the presence and absence of individual genes associated with each locus based on PyParanoid presence-absence data. Coloured squares represent the presence of a homologous gene, while absence is represented by white. The tree exclusively shows the *P. fluorescens* subgroups *P. protegens* and *P. chlororaphis*.

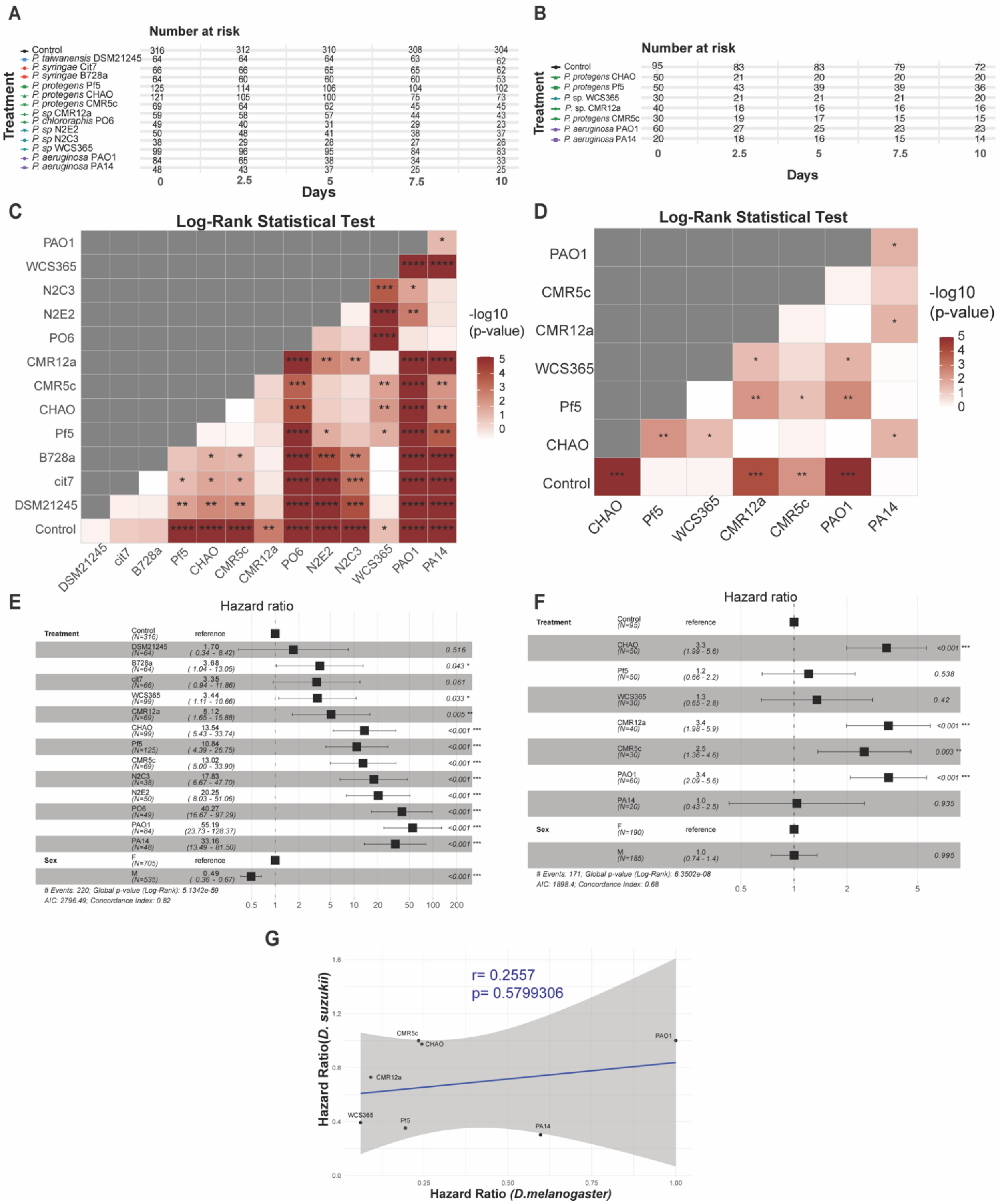

Figure S3. Log-rank tests and Hazard ratios from Cox proportional hazards model. (A–B) Number of flies at risk per day for each treatment shown in Figure 2, used to generate Kaplan–Meier survival curves. (C–D) Pairwise Mantel–Cox (log-rank) tests between infection conditions. Significance: $p \leq 0.05$ (*), $p \leq 0.01$ (**), $p \leq 0.001$ (***) and $p \leq 0.0001$ (****). Blank squares indicate non-significant comparisons ($p > 0.05$); grey squares indicate non-applicable comparisons from Figure 2. Forest plots display hazard ratios (HR) with 95%

confidence intervals for each bacterial treatment; significance levels are indicated by asterisks. (E) *Drosophila melanogaster* survival experiments. (F) *Drosophila suzukii* survival experiments. (G) Linear regression of *D. melanogaster* and *D. suzukii* hazard ratios per strain. Shaded region shows the 95% confidence interval for the regression (blue line), with Pearson's correlation coefficient (*r*) and *p*-value.

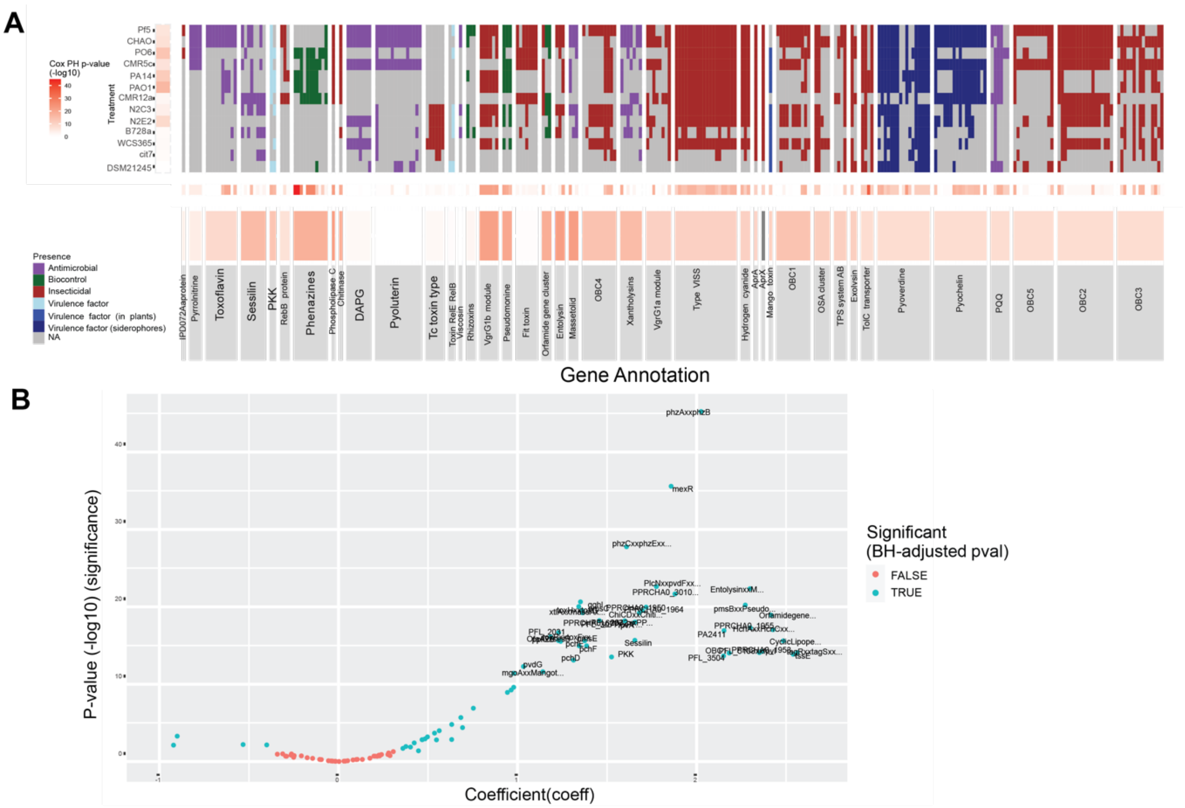

Figure S4. Analysis of genes associated with *D. melanogaster* survival

A) Cox proportional hazard and random forest models were used to predict the survival of *D. melanogaster* based on the presence or absence of specific genes. The *p*-values from the Cox proportional hazards model are represented by the red boxes, indicating the potential association between the presence of a particular gene and the risk of mortality in flies. The red boxes on the y-axis explain the *p*-value of survival probability after inoculation with each strain. The red boxes on the top horizontal row show the individual gene identity risk *p*-value, while the bottom horizontal row shows the *p*-value for gene annotation. The presence of genes is colour-coded based on their specific functions from the literature. B) Volcano plot of the genes based on *p*-value and coefficient. Significant genes are labeled in blue.

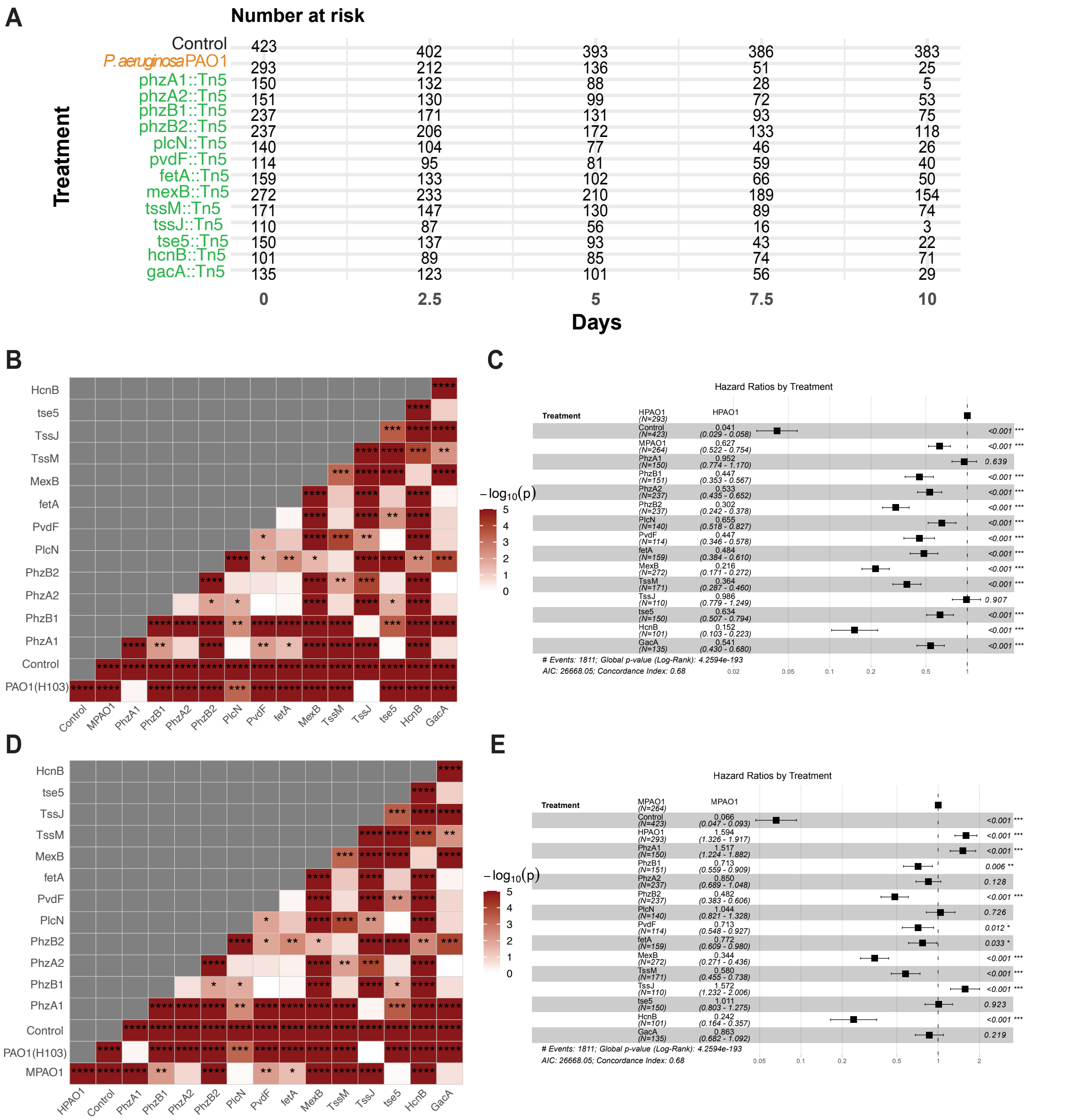

Figure S5. Pairwise Mantel–Cox (log-rank) tests and Forest plots of Hazard ratios comparing survival curves from the Kaplan–Meier experiments shown in Figure 5. A) Number of flies at risk per day for each treatment shown in Figure 5, used to generate Kaplan–Meier survival curves. (B-C) Comparisons of transposon mutants against the reference *P. aeruginosa* PAO1 (H103). (D-E) Comparisons of transposon mutants and PAO1 H103 against the reference *P. aeruginosa* PAO1 (MPAO1). (B-E) Statistical significance is denoted as $p \leq 0.05$ (*), $p \leq 0.01$ (**), and $p < 0.001$ (***) and $p < 0.0001$ (****). Blank squares indicate non-significant comparisons ($p > 0.05$), and grey squares represent comparisons that were not applicable.

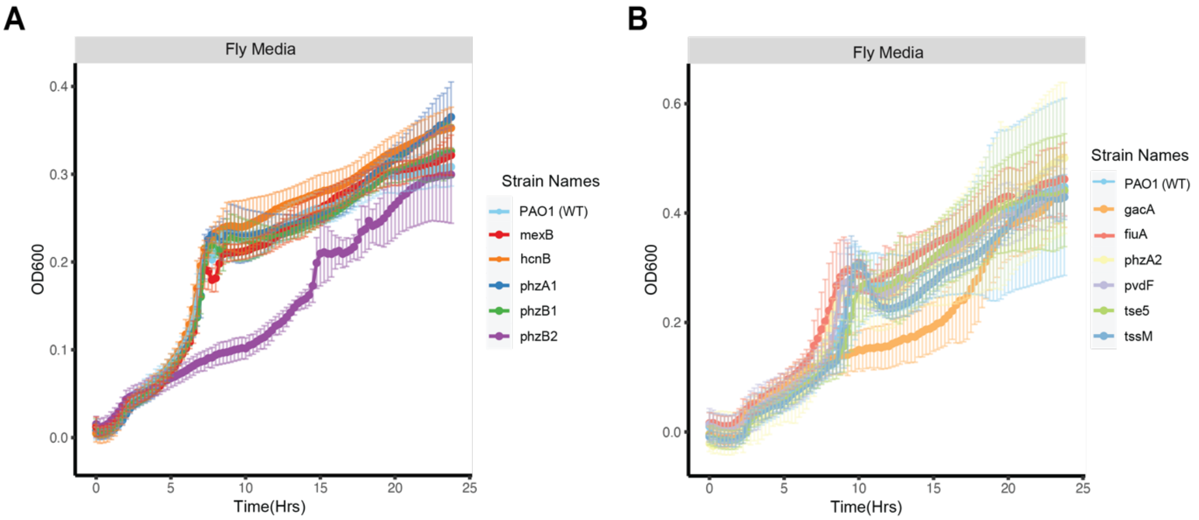

Figure S6. Growth curves in M9 medium supplemented with fly extract. Bacterial strains were grown in M9 medium supplemented with fly extract. A) Growth comparison using batch 1 fly extract, assessing PAO1 wild type against transposon mutants *mexB*, *hcnB*, *phzA1*, *phzB1*, and *phzB2*. B) Growth comparison using batch 2 fly extract, assessing PAO1 wild type against transposon mutants *gacA*, *fiuA*, *phzA2*, *pvdF*, *tse5*, and *tssM*.

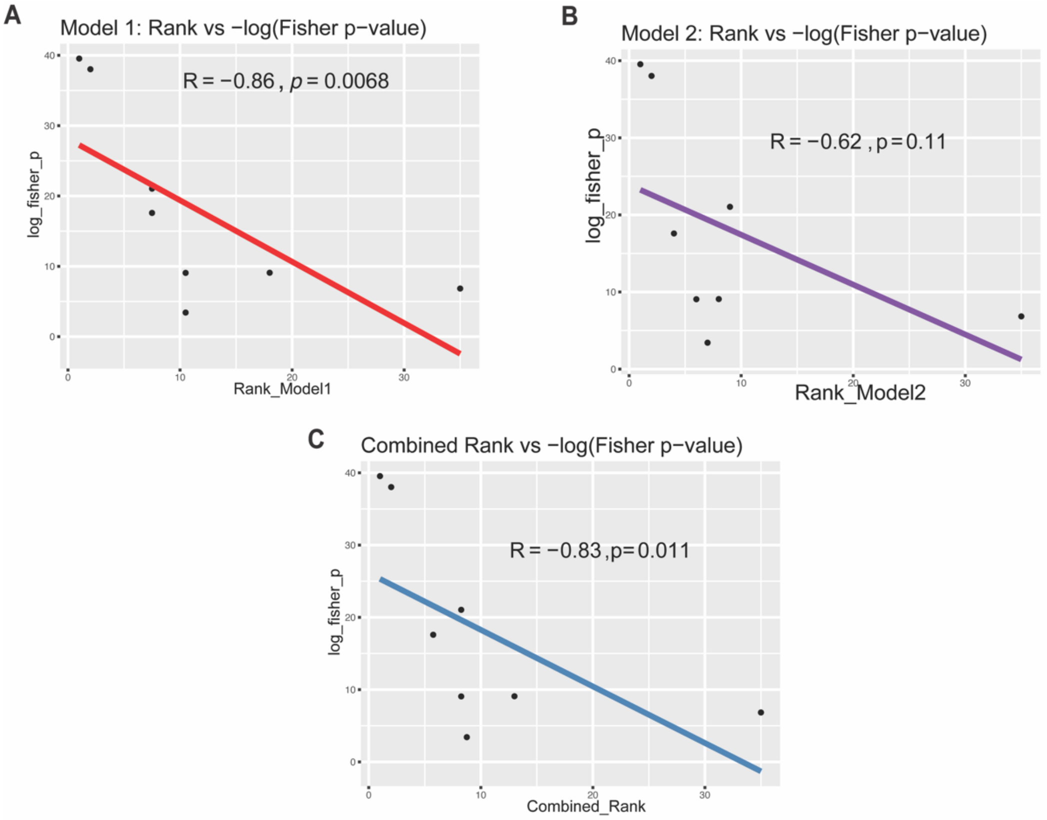

Figure S7. Comparison of model-based gene rankings with experimental survival data. (A–C) Spearman correlations between model rankings and the aggregated survival significance of each gene, represented by -log (10) Fisher's combined *p*-value from Kaplan–Meier survival curves (Figure 5). Higher -log (10) values indicate stronger evidence of reduced fly survival upon infection with the corresponding mutant strain. Each point corresponds to a gene, and regression lines (red, purple, and blue) indicate trends for Model 1 (A), Model 2 (B), and the combined ranking (C).

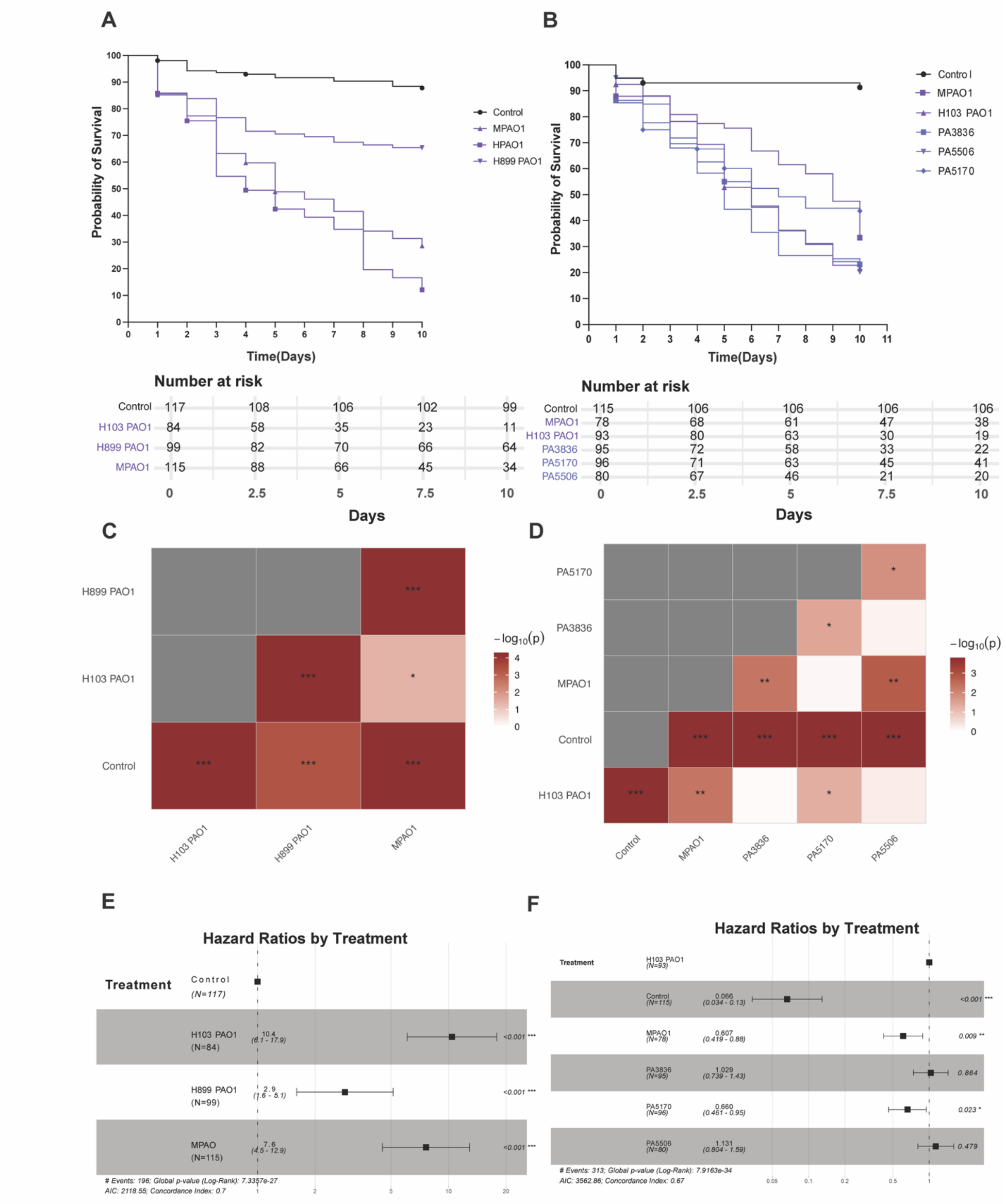

Figure S8. Testing the virulence of *P. aeruginosa* parental strains. A) Kaplan-Meier (KM) survival curves of *D. melanogaster* Oregon-R flies following oral infection with *Pseudomonas* aeruginosa PA01 (MPAO1, H103PAO1 and H899PAO1) (OD600 = 100) or control 5% sucrose solution. B) Kaplan-Meier (KM) survival curves of *D. melanogaster* Oregon-R flies following oral infection with *Pseudomonas* aeruginosa PA01 (MPAO1 and H103PAO1), as well as the non-coding mutations PA3836, PA5506, PA5170 (OD600 = 100) or control 5% sucrose solution.

Each replicate included at least 20 flies per experiment. C and D) Pairwise Mantel–Cox (log-rank) tests were performed between infection conditions. Significance is shown as $p \leq 0.05$ (*), $p \leq 0.01$ (**), $p \leq 0.001$ (***). Blank squares represent non-significant comparisons ($p > 0.05$); grey squares indicate comparisons that were not applicable. E and F) Forest plots displaying the hazard ratio (HR) and 95% confidence intervals for each bacterial treatment. Asterisks indicate the significance level.

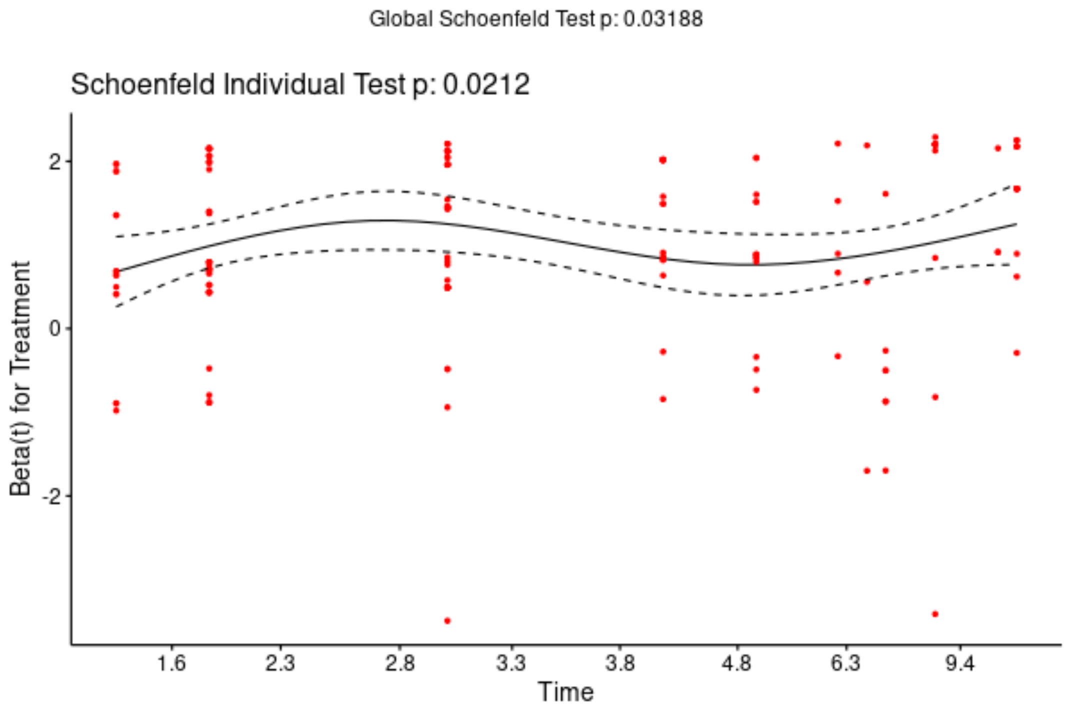

Figure S9. Schoenfeld residuals for Cox models of fly mortality. The y-axis shows the time-varying coefficient β(t) for the treatment effect, and the x-axis shows time. The central curve is the estimated β(t), with dashed lines indicating approximate 95% confidence intervals. Proportional hazards assumptions were tested using Schoenfeld residuals (`cox.zph`), with individual $p = 0.0212$ and global $p = 0.0319$. Minor deviations were observed, but residuals showed no major violations, supporting the Cox model as a screening tool for candidate genes associated with fly mortality.

Supplementary References

1. Flury P, Vesga P, Péchy-Tarr M, Aellen N, Dennert F, Hofer N, Kupferschmied KP, Kupferschmied P, Metla Z, Ma Z, Siegfried S, de Weert S, Bloemberg G, Höfte M, Keel CJ, Maurhofer M. 2017. Antimicrobial and Insecticidal: Cyclic Lipopeptides and Hydrogen Cyanide Produced by Plant-Beneficial Pseudomonas Strains CHA0, CMR12a, and PCL1391 Contribute to Insect Killing. Front Microbiol 8:100.

2. Ruffner B, Péchy-Tarr M, Ryffel F, Hoegger P, Obrist C, Rindlisbacher A, Keel C, Maurhofer M. 2013. Oral insecticidal activity of plant-associated pseudomonads. Environmental Microbiology 15:751–763.

3. Carlson AB, Mathesius CA, Ballou S, Boeckman CJ, Gunderson TA, Mirsky HP, Mukerji P, Roe JC, Schmidt JM, Zhang J, Delaney B. 2019. Safety assessment of coleopteran active IPD072Aa protein from Pseudomonas chlororaphis. Food and Chemical Toxicology 129:376–381.

4. Opota O, Vallet-Gély I, Vincentelli R, Kellenberger C, Iacovache I, Gonzalez MR, Roussel A, van der Goot F-G, Lemaitre B. 2011. Monalysin, a novel ß-pore-forming toxin from the Drosophila pathogen Pseudomonas entomophila, contributes to host intestinal damage and lethality. PLoS Pathog 7:e1002259.

5. Flury P, Aellen N, Ruffner B, Péchy-Tarr M, Fataar S, Metla Z, Dominguez-Ferreras A, Bloemberg G, Frey J, Goesmann A, Raaijmakers JM, Duffy B, Höfte M, Blom J, Smits THM, Keel C, Maurhofer M. 2016. Insect pathogenicity in plant-beneficial pseudomonads: phylogenetic distribution and comparative genomics. ISME J 10:2527–2542.

6. Lee SA, Jang SH, Kim BH, Shibata T, Yoo J, Jung Y, Kawabata S, Lee BL. 2018. Insecticidal activity of the metalloprotease AprA occurs through suppression of host cellular and humoral immunity. Developmental and Comparative Immunology 81:116–126.

7. Job V, Bouillot S, Gueguen E, Robert-Genthon M, Panchev P, Elsen S, Attrée I. 2019. Pseudomonas two-partner secretion toxin Exolysin contributes to insect killing. bioRxiv https://doi.org/10.1101/807867.

8. Olorunleke FE, Kieu NP, De Waele E, Timmerman M, Ongena M, Höfte M. 2017. Coregulation of the cyclic lipopeptides orfamide and sessilin in the biocontrol strain Pseudomonas sp. CMR12a. MicrobiologyOpen 6:e00499.

9. Loper JE, Henkels MD, Rangel LI, Olcott MH, Walker FL, Bond KL, Kidarsa TA, Hesse CN, Sneh B, Stockwell VO, Taylor BJ. 2016. Rhizoxin analogs, orfamide A and chitinase production contribute to the toxicity of Pseudomonas protegens strain Pf-5 to Drosophila melanogaster. Environmental Microbiology 18:3509–3521.

10. Li W, Rokni-Zadeh H, De Vleeschouwer M, Ghequire MGK, Sinnaeve D, Xie G-L, Rozenski J, Madder A, Martins JC, De Mot R. 2013. The antimicrobial compound xantholysin defines a new group of Pseudomonas cyclic lipopeptides. PLoS One 8:e62946.

11. Chen Y, Shen X, Peng H, Hu H, Wang W, Zhang X. 2015. Comparative genomic analysis and phenazine production of Pseudomonas chlororaphis, a plant growth-promoting rhizobacterium. Genom Data 4:33–42.

12. Vesga P, Flury P, Vacheron J, Keel C, Croll D, Maurhofer M. 2020. Transcriptome plasticity underlying plant root colonization and insect invasion by Pseudomonas protegens. 11. ISME J 14:2766–2782.

13. Raymann K, Bobay L-M, Doak TG, Lynch M, Gribaldo S. 2013. A Genomic Survey of Reb Homologs Suggests Widespread Occurrence of R-Bodies in Proteobacteria. G3 (Bethesda) 3:505–516.

14. Vacheron J, Péchy-Tarr M, Brochet S, Heiman CM, Stojiljkovic M, Maurhofer M, Keel C. 2019. T6SS contributes to gut microbiome invasion and killing of an herbivorous pest insect by plant-beneficial Pseudomonas protegens. ISME J 13:1318–1329.

15. Ma M, Lustig M, Salem M, Mengin-Lecreulx D, Phan G, Broutin I. 2021. MexAB-OprM Efflux Pump Interaction with the Peptidoglycan of Escherichia coli and Pseudomonas aeruginosa. 10. International Journal of Molecular Sciences 22:5328.

16. Gu Q, Qiao J, Wang R, Lu J, Wang Z, Li P, Zhang L, Ali Q, Khan AR, Gao X, Wu H. 2022. The Role of Pyoluteorin from Pseudomonas protegens Pf-5 in Suppressing the Growth and Pathogenicity of Pantoea ananatis on Maize. Int J Mol Sci 23:6431.

17. Pawar S, Chaudhari A, Prabha R, Shukla R, Singh DP. 2019. Microbial Pyrrolnitrin: Natural Metabolite with Immense Practical Utility. Biomolecules 9:443.

18. de Bruijn I, de Kock MJD, de Waard P, van Beek TA, Raaijmakers JM. 2008. Massetolide A Biosynthesis in Pseudomonas fluorescens. Journal of Bacteriology 190:2777–2789.

19. Alsohim AS, Taylor TB, Barrett GA, Gallie J, Zhang X-X, Altamirano-Junqueira AE, Johnson LJ, Rainey PB, Jackson RW. 2014. The biosurfactant viscosin produced by Pseudomonas fluorescens SBW25 aids spreading motility and plant growth promotion. Environmental Microbiology 16:2267–2281.

20. Rashid MH, Rumbaugh K, Passador L, Davies DG, Hamood AN, Iglewski BH, Kornberg A. 2000. Polyphosphate kinase is essential for biofilm development, quorum sensing, and virulence of Pseudomonas aeruginosa. Proceedings of the National Academy of Sciences 97:9636–9641.

21. Rangel LI, Henkels MD, Shaffer BT, Walker FL, Davis EW, Stockwell VO, Bruck D, Taylor BJ, Loper JE. 2016. Characterization of Toxin Complex Gene Clusters and Insect Toxicity of Bacteria Representing Four Subgroups of Pseudomonas fluorescens. PLoS One 11:e0161120.

22. Faucher SP, Matthews S, Nickzad A, Vounba P, Shetty D, Bédard É, Prévost M, Déziel E, Paranjape K. 2022. Toxoflavin secreted by Pseudomonas alcaliphila inhibits the growth of Legionella pneumophila and Vermamoeba vermiformis. Water Res 216:118328.

23. Mercado-Blanco J, van der Drift KM, Olsson PE, Thomas-Oates JE, van Loon LC, Bakker PA. 2001. Analysis of the pmsCEAB gene cluster involved in biosynthesis of salicylic acid and the siderophore pseudomonine in the biocontrol strain Pseudomonas fluorescens WCS374. J Bacteriol 183:1909–1920.

24. Lim CK, Penesyan A, Hassan KA, Loper JE, Paulsen IT. 2016. Disruption of Transporters Affiliated with Enantio-Pyochelin Biosynthesis Gene Cluster of Pseudomonas protegens Pf-5 Has Pleiotropic Effects. PLoS One 11:e0159884.

25. Liu Y, Dai C, Zhou Y, Qiao J, Tang B, Yu W, Zhang R, Liu Y, Lu S-E. 2021. Pyoverdines Are Essential for the Antibacterial Activity of Pseudomonas chlororaphis YL-1 under Low-Iron Conditions. Applied and Environmental Microbiology 87:e02840-20.

26. Misra HS, Rajpurohit YS, Khairnar NP. 2012. Pyrroloquinoline-quinone and its versatile roles in biological processes. J Biosci 37:313–325.

27. Mahmoudi M, Sadeghifard N, Maleki A, Yeo CC, Ghafourian S. 2022. relBE toxin-antitoxin system as a reliable anti-biofilm target in Pseudomonas aeruginosa. J Appl Microbiol 133:683–695.

28. Vallet-Gely I, Novikov A, Augusto L, Liehl P, Bolbach G, Péchy-Tarr M, Cosson P, Keel C, Caroff M, Lemaitre B. 2010. Association of hemolytic activity of Pseudomonas entomophila, a versatile soil bacterium, with cyclic lipopeptide production. Appl Environ Microbiol 76:910–921.

29. Carrión VJ, van der Voort M, Arrebola E, Gutiérrez-Barranquero JA, de Vicente A, Raaijmakers JM, Cazorla FM. 2014. Mangotoxin production of Pseudomonas syringae pv. syringae is regulated by MgoA. BMC Microbiology 14:46.
